# The EMPOWER-SUSTAIN e-Health Intervention to improve patient activation and self-management behaviours among individuals with Metabolic Syndrome in primary care: study protocol for a pilot randomised controlled trial

**DOI:** 10.1186/s13063-020-04237-x

**Published:** 2020-04-05

**Authors:** Maryam Hannah Daud, Anis Safura Ramli, Suraya Abdul-Razak, Mohamad Rodi Isa, Fakhrul Hazman Yusoff, Noorhida Baharudin, Mohamed Syarif Mohamed-Yassin, Siti Fatimah Badlishah-Sham, Azlina Wati Nikmat, Nursuriati Jamil, Hapizah Mohd-Nawawi

**Affiliations:** 1 Institute of Pathology, Laboratory and Forensic Medicine (I-PPerForM), Universiti Teknologi MARA, Sungai Buloh Campus, Jalan Hospital, 47000 Sungai Buloh, Selangor Malaysia; 2 Department of Primary Care Medicine, Faculty of Medicine, Universiti Teknologi MARA, Selayang Campus, 68100 Batu Caves, Selangor Malaysia; 3 Department of Population Health & Preventive Medicine, Faculty of Medicine Universiti Teknologi MARA, Sungai Buloh Campus, Jalan Hospital, 47000 Sungai Buloh, Selangor Malaysia; 4 Department of Computer Science, Faculty of Computer and Mathematical Sciences, Universiti Teknologi MARA, 40450 Shah Alam, Selangor Malaysia; 5 Department of Psychiatry, Faculty of Medicine, Universiti Teknologi MARA, Sungai Buloh, Campus, Jalan Hospital, 47000 Sungai Buloh, Selangor Malaysia

**Keywords:** E-health intervention, Self-management, Patient activation, Chronic Care Model, Chronic disease management, Multifaceted intervention, Metabolic syndrome, Primary care

## Abstract

**Background:**

Epidemiological studies conducted in various parts of the world have clearly demonstrated that metabolic syndrome (MetS) is an increasing global health problem, not only in Western societies but also in Asian populations. Web-based and mobile phone-based self-management applications have been proven to be effective in improving self-management behaviour of patients with MetS components (i.e., diabetes or hypertension). However, evidence is lacking in terms of their effectiveness specifically for patients with MetS. The aim of this pilot study is to evaluate the feasibility and potential effectiveness of the EMPOWER-SUSTAIN Self-Management e-Health Intervention in improving activation and self-management behaviours among patients with MetS. This paper presents the study protocol.

**Methods:**

A pilot randomised controlled trial will be conducted in a university primary care clinic. A total of 232 patients aged 18–60 years with MetS will be recruited; 116 will be randomised to receive the EMPOWER-SUSTAIN intervention for 6 months, and another 116 patients will continue with usual care. The EMPOWER-SUSTAIN intervention is a multifaceted chronic disease management strategy based on the Chronic Care Model and persuasive technology theory. It consists of training primary care physicians, nurses and patients to use the EMPOWER-SUSTAIN web-based self-management mobile app, strengthening the patient–physician relationship and reinforcing the use of relevant clinical practice guidelines to guide management and prescribing. The primary outcome is the mean change in patient activation score using the Patient Activation Measure short form Malay version (PAM-13-M) questionnaire. The secondary outcomes include the changes in waist circumference, body mass index, blood pressure, patient physical activity level, eating behaviour, perception of chronic illness care, satisfaction with patient–physician interaction, and perceived absolute 10-year cardiovascular disease risk. Feasibility of implementing the intervention will be evaluated. This includes acceptability of the intervention, estimating the likely rate of participant recruitment and retention, appropriateness of the outcome measures, calculation of sample size, and the intervention’s potential effectiveness.

**Conclusion:**

To our knowledge, this is the first study in Malaysia that aims to determine the feasibility of a multifaceted e-health intervention, as well as to indicate more useful aspects of this intervention for further exploration in a larger trial.

**Trial registration:**

ClinicalTrials.gov, NCT04120779. Registered on 9 October 2019, protocol version 1.

## Background

According to the International Diabetes Federation (IDF) consensus worldwide definition of metabolic syndrome (MetS), it is estimated that around 20–25% of the world’s adult population have MetS [1, 2]. The cardiovascular disease (CVD) risk factors tend to cluster in an individual, giving rise to MetS, which is defined by the presence of central obesity, elevated blood pressure (BP), elevated plasma glucose, and dyslipidaemia [3]. The prevalence of MetS ranges from 11.9% to 37.1% in the Asia-Pacific region, based on a systematic review [4]. These include the Philippines (11.9%), China (21.3%), Sri Lanka (24.3%), Taiwan (25.5%), Singapore (26.9%), South Korea (31.3%), Mongolia (32.8%) and Malaysia (37.1%) [4]. In Malaysian adults ≥30 years old, the prevalence of MetS was found to be 43.4% [5]. A recent review showed that MetS affected 25–44% of the adult population in Malaysia, with the risk increasing with age [6].

The prevalence of MetS has reached epidemic proportions in many Asian countries, including Malaysia, especially in the younger generations [7, 8]. Rapid economic growth, socio-demographic change, and adoption of unhealthy lifestyle that have all occurred over the past few decades are thought to be responsible for this rising prevalence [9]. This in turn has resulted in an upsurge of CVD morbidity and mortality in Malaysia [9]. A recent national report showed that among 95% of patients presenting with acute coronary syndrome (ACS), 46.2% had diabetes, 64% had hypertension and 38.6% had dyslipidaemia [10]. Malaysians were found to develop ACS at a younger age than their Asian and Western counterparts [10]. The mean age of individuals with ACS at admission in Malaysia was 58.6 years old, 23.6% of whom were under the age of 50 years [10].

One of the promising approaches to improve management of MetS in primary care is the integration of the Chronic Care Model (CCM) into the health system. The CCM is the best-known model for transforming chronic disease management in primary care [11]. It focuses on linking informed, activated patients with proactive and prepared health care teams [11, 12]. CCM offers effective strategies to improve chronic disease outcomes in primary care [12, 13]. A series of recent studies have indicated that CCM improves the quality of care and outcomes for patients with various chronic conditions [14–16]. In Malaysia, our previous work has shown that it was feasible to implement the CCM in the primary care setting [17]. In the EMPOWER-PAR Study, we have shown that the implementation of at least three components of CCM, which include self-management support and improved glycaemic control among patients with type 2 diabetes mellitus [18].

One of the most essential components of the CCM is self-management support, which encompasses activities that empower patients to become activated to manage their own health [11, 12]. International clinical guidelines recommend the inclusion of self-management programs in the management of MetS and the associated cardiovascular (CV) risk factors, and such programs have been associated with improved health outcomes [12, 19]. A systematic review of 19 studies reveals that self-management intervention improved outcomes, including haemoglobin A1c (HbA1c), waist circumference (WC), self-efficacy and empowerment in patients with MetS [20]. Lifestyle modification intervention has been shown to be effective in improving fasting blood glucose (FBG), WC, BP and triglycerides (TG) in patients with MetS [21].

Conceptually, self-management has a close association with patient activation and empowerment [22, 23]. Patient activation reflects patients’ central role in having the knowledge, skills and confidence to make effective decisions to self-manage their own health and change their behaviour (e.g., in adopting a healthy lifestyle) [23–26]. It is considered to be the most reliable indicator of the willingness and ability to manage health autonomously [27–29]. Previous studies have emphasised that increased patient activation level has been shown to improve self-management behaviours, such as physical activity (PA), diet and medication adherence, in patients with various chronic conditions [25, 30–32]. Patient activation has also been shown to improve quality of life and health outcomes and to reduce the use of health care services and costs for patients with various chronic conditions [25, 30–33]. However, there is a gap in the literature pertaining to intervention targeting to improve patient activation in patients with multiple CVD risk factors, such as MetS, especially in the younger generations.

To meet the growing demands of the younger generation, efforts have been made to develop web or mobile applications for self-management of multiple CV risk factors [34–38]. To ensure sustainability, the application should be developed using an evidence-based approach such as persuasive technology [39]. This technology was established to motivate people by influencing attitudes and behaviours of the users through persuasion and social influence, but not through pressure or force [39].

However, to our knowledge, there is no e-health self-management system which has been developed to suit the younger generations with MetS in Malaysia. Therefore, the objective of this study is to evaluate the feasibility and potential effectiveness of the EMPOWER-SUSTAIN Self-Management e-Health Intervention (multifaceted strategies involving web-based self-management mobile apps based on the CCM and persuasive technology theory) in improving patient activation and self-management behaviours compared with usual care among individuals with MetS in the Malaysian primary care setting. This paper describes the design of the trial as well as the development of the EMPOWER-SUSTAIN Self-Management e-Health Intervention and its underpinning conceptual frameworks.

### Hypotheses

The primary hypothesis is that the mean score of patient activation would improve with the EMPOWER-SUSTAIN Self-Management e-Health Intervention. The secondary hypotheses are that WC, body mass index (BMI), BP, PA level, eating behaviour, patients’ perceptions and experiences of receiving care for chronic conditions, patient–physician satisfaction, and the accuracy of the CVD risk perception would improve with the EMPOWER-SUSTAIN Self-Management e-Health Intervention.

### Trial design

This is a pilot randomised controlled trial with parallel group design (i.e., intervention vs. control [usual care] with an allocation ratio of 1:1). The overall duration of the study is 1 year, and the duration of the intervention is 6 months. Blinding is not possible, owing to the nature and complexity of the intervention.

## Methods

### Protocol registration

**Fig. 1:**
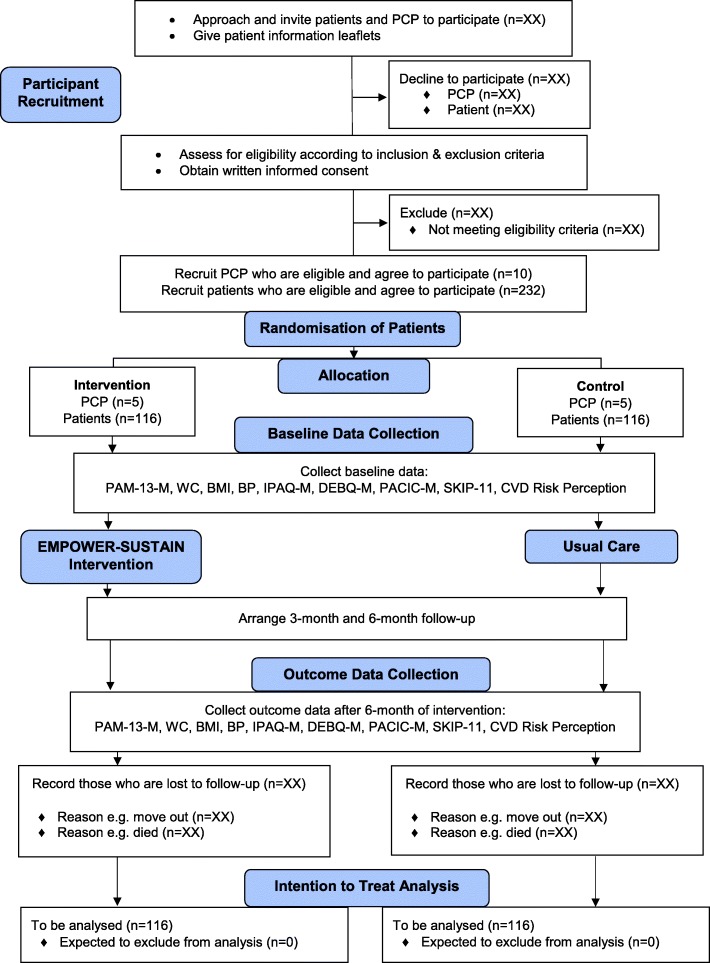
The EMPOWER-SUSTAIN Consolidated Standards of Reporting Trials (CONSORT) flow diagram

The study protocol is registered with ClinicalTrials.gov (identifier NCT04120779), and the registration complied with all the items in the World Health Organisation Trial Registration Data Set. This is the first version of the study protocol. The reporting of this paper is done in accordance with the Standard Protocol Items: Recommendations for Interventional Trials (SPIRIT) 2013 guidance and checklist for protocols of clinical trials [40] and the Consolidated Standards of Reporting Trials (CONSORT) checklist for pilot and feasibility trials [41]. The SPIRIT checklist is provided in Additional file 1. Figure 1 shows the EMPOWER-SUSTAIN CONSORT flow diagram [41].

### Study setting

This pilot study will be conducted at a university primary care clinic, which is located in the state of Selangor, Malaysia. It is a busy primary care clinic with a load of approximately 500 patients per day. Almost 70% of the patients are under regular follow-up at this clinic for various long-term conditions, including MetS.

The pilot randomised controlled trial [42] is conducted to ensure that the intervention can be delivered as intended and that safe assumptions can be made about effect size, rate of recruitment and retention in the future definitive clinical trial [43]. In this pilot study, the feasibility of implementing the EMPOWER-SUSTAIN Self-Management e-Health Intervention for patients with MetS in a primary care clinic will be evaluated. These include acceptability of the intervention, estimating the likely rate of participant recruitment and retention, appropriateness of the outcome measures, calculation of sample size, and its potential effectiveness (i.e., the effect size) [44, 45].

### Study population

The study population will comprise individuals aged 18 to 60 years old who are diagnosed with MetS according to the Joint Interim Statement (JIS) on MetS definition of 2009 issued by the IDF Task Force on Epidemiology and Prevention; National Heart, Lung, and Blood Institute; American Heart Association; World Heart Federation; International Atherosclerosis Society; and International Association for the Study of Obesity [1].

According to the JIS definition [1], MetS is defined by the presence of at least three of five of the following risk factors:

1. WC: males ≥ 90 cm, females ≥ 80 cm (South Asian cut-points)
2. BP: systolic BP ≥ 130 mmHg and/or diastolic BP ≥ 85 mmHg or receiving treatment for hypertension (HPT)
3. FBG: ≥ 5.6 mmol/L or receiving treatment for elevated glucose
4. TG: ≥ 1.7 mmol/L or receiving treatment for TG
5. High-density lipoprotein cholesterol (HDL-c): males < 1.0 mmol/L, females < 1.3 mmol/L or receiving treatment for HDL-c

### Patient recruitment

Consecutive patients who attend the university primary care clinic during the recruitment period will be approached, given the patient information sheet about the study, and invited to participate. Those who are willing to participate will be interviewed and screened by the investigators to identify eligibility on the basis of inclusion and exclusion criteria. Written informed consent will be obtained from those who are eligible, and they will be recruited into the study.

### Inclusion criteria

Patients 18 to 60 years old who fulfil all of the following inclusion criteria will be included:

1. Diagnosed with MetS according to the JIS definition [1]
2. Have received follow-up care for MetS at the university primary care clinic at least twice in the last 1 year
3. Have regular access to the internet
4. Perceive that they have basic skills to use the web and smart mobile phone
5. Are able to read and understand written English or Malay

### Exclusion criteria

Patients who fulfil any of the following criteria will be excluded:

1. Type 1 diabetes mellitus
2. Receiving renal dialysis
3. Present with severe hypertension (systolic BP > 180 mmHg and/or diastolic BP > 110 mmHg) at recruitment
4. Diagnosed with conditions resulting in secondary hypertension
5. Diagnosed with circulatory disorders requiring referral to secondary care over the last 1 year and during the course of the study (e.g., unstable angina, heart attack, stroke, transient ischaemic attacks, peripheral vascular diseases)
6. Receiving shared care at primary and secondary care centres for CVD (coronary artery diseases, stroke, transient ischaemic attacks, peripheral vascular diseases)
7. Receiving chemotherapy/radiotherapy or palliative care
8. Diagnosed with a psychiatric illness such as schizophrenia, bipolar disorder, major depression
9. Diagnosed with cognitive impairment such as dementia
10. Pregnant
11. Enrolled in another intervention study

### Physician recruitment

All primary care physicians (PCPs) who are practicing at the university primary care clinic will be invited to participate in the study. To be eligible, the following criteria must be met:

1. Have one or more years of working experience in a primary care setting
2. Must be keen to participate in the study
3. Willing to deliver the EMPOWER-SUSTAIN e-Health Intervention to patients with MetS

At least ten PCPs who meet the eligibility criteria will be recruited. Five PCPs will be allocated to deliver the EMPOWER-SUSTAIN intervention, and the rest of the PCPs will continue with usual care.

### EMPOWER-SUSTAIN Self-Management e-Health Intervention

**Fig. 2:**
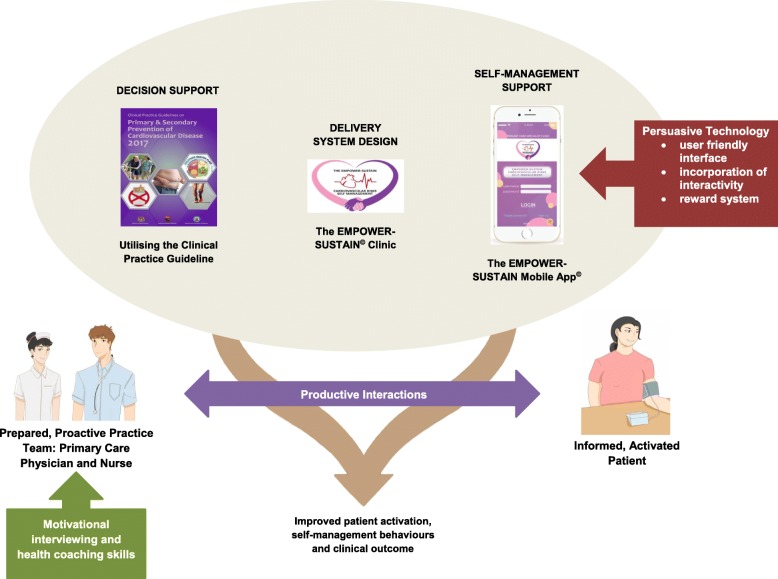
The conceptual framework for the EMPOWER-SUSTAIN Self-Management e-Health Intervention

The EMPOWER-SUSTAIN Self-Management e-Health Intervention is a complex intervention involving multifaceted components using CCM as the conceptual framework [11, 12]. This is in line with the United Kingdom Medical Research Council (MRC) complex intervention framework, which recommends that intervention development be guided by best available evidence and an appropriate framework [46, 47]. A comprehensive review of the literature was conducted to identify the crucial components of the EMPOWER-SUSTAIN intervention that could improve patient activation and self-management behaviours. Three crucial components were identified on the basis of three CCM elements (i.e., delivery system design, self-management support and decision support) and will be included in the EMPOWER-SUSTAIN Self-Management e-Health Intervention as described in Table 1. Figure 2 illustrates the conceptual framework underpinning the EMPOWER-SUSTAIN Self-Management e-Health Intervention.

**Table 1:** EMPOWER-SUSTAIN Self-Management e-Health Intervention

Chronic Care Model elements	Crucial components	Intervention
Decision support	Decision support for physicians to translate CPG recommendations into day-to-day clinical practice [48–50]	EMPOWER-SUSTAIN Training Workshop for PCPs to use web-based desktop and mobile applications and to reinforce the use of relevant CPG for management and prescribing
Self-management support	Self-management support to facilitate patient activation and behaviour change [51–54]	PCPs to support and empower patients with knowledge, skills and confidence in using the EMPOWER-SUSTAIN mobile application and the EMPOWER-SUSTAIN Global CV Risks Self-Management Booklet using the persuasive technology approach
Delivery system design	Delivery system design through the EMPOWER-SUSTAIN clinic to improve continuity of care and patient–physician relationship [55–57]	The EMPOWER-SUSTAIN Self-Management e-Health Intervention will be delivered by PCP at baseline and 3-month and 6-month follow-up visits in the EMPOWER-SUSTAIN clinic to ensure continuity of care and sustainability

*CPG* Clinical practice guidelines, *PCP* Primary care physician

### Development of the EMPOWER-SUSTAIN self-management mobile application

The EMPOWER-SUSTAIN web-based self-management mobile application is currently at the final stage of development. The iterative model was chosen as the software development model for this study [58]. Content from the newly revised EMPOWER-SUSTAIN Global Cardiovascular Risks Self-Management Booklet was evaluated for its suitability to be included in the prototype. A storyboard was designed to create the flow of prototype use by PCPs, nurses and patients during follow-up in the clinic and at home. In the pre-alpha stage, wireframe was designed to describe and visualise the user interface in static draft layouts based on the content and structure of information. Based on the wireframe, high-fidelity mock-up static graphic diagrams demonstrating the content and function of the prototype divided into eight sections were designed. The sections included My Profile, My Cardiovascular Risks, My Treatment Targets, My Check-Up, My Weight Management, My Smoking Habit, My Self-Management, and My Medication. Persuasive technology theory [39, 59] was used to build the evidence-based content, which is presented in a user-friendly interface, with incorporation of interactivity and a reward system. This app is designed to aid and motivate people to adopt a positive attitude and behaviour change through persuasion and a reward system. The EMPOWER-SUSTAIN mock prototype mobile app is provided in Additional file 2. Based on the mock graphic diagrams, a working prototype of the EMPOWER-SUSTAIN self-management mobile app was developed using the iterative model of the Software Development Life Cycle [58, 60]. A complementary desktop application is also being developed for the PCPs and nurses to use in the clinic. This prototype is currently undergoing alpha (utility) testing by medical experts [61, 62] and beta (usability) testing by patients with MetS [63]. Data which are entered by the PCPs and patients into the app will be stored in a secure server. The feasibility of using the app will be evaluated in this pilot study.

### Conduct of the EMPOWER-SUSTAIN Workshop

Prior to the delivery of the intervention, the five PCPs in the intervention arm and a nurse will be trained on how to use the EMPOWER-SUSTAIN desktop and mobile applications during the EMPOWER-SUSTAIN Workshop. Training will also include reinforcing the use of relevant clinical practice guidelines (CPG) [64] as decision support for management and prescribing. The PCPs and nurse will be trained on the basis of Self-Determination Theory (SDT), which would be useful for them to understand how patients’ behaviour change can be influenced by health care providers [65]. They will also be trained in motivational interview (MI) techniques and health-coaching skills to facilitate patients’ autonomous self-regulation to enhance their behaviour change [65–68]. MI training would include the skills to formulate open-ended questions, reflective statements (i.e., restating what the patients conveyed) and stimulating what the patients know before providing relevant education [65]. The main aim of the MI is to engage patients in problem solving by encouraging active learning [66] and facilitating patients’ autonomous self-regulation to achieve goals by enabling patients to develop individualised action plans [67, 68].

### Delivery of the intervention

**Fig. 3:**
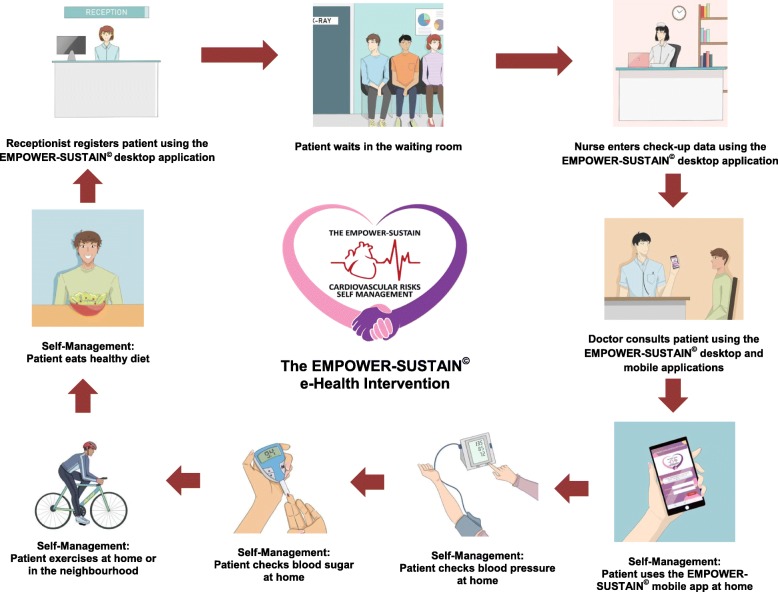
Delivery of the EMPOWER-SUSTAIN Self-Management e-Health Intervention

The EMPOWER-SUSTAIN Self-Management e-Health Intervention will be professionally delivered to the individual patients by the PCPs, assisted by a nurse. Patients will be given the EMPOWER-SUSTAIN self-management app, which will be downloaded to their mobile phone. Follow-up care by the PCPs will be arranged at baseline, 3 months and 6 months. At baseline, patients in the intervention arm will be given a username and password to access the app. They will be trained individually on its use by the PCPs assisted by a nurse using the Knowledge to Action (KTA) Framework [69] and the persuasive technology theory [39, 59]. The KTA Framework incorporates the need to adapt the knowledge to fit with individual context. In addition to this, PCPs and the nurse will provide problem-solving assistance and support for patients to use the app in line with the theory of persuasive technology. MI techniques and health-coaching skills to facilitate patients’ autonomous self-regulation to enhance their behaviour change will also be applied. PCPs and the nurse will go through each section with the patients and will ensure that information recorded in all sections is complete. Patients will be guided to navigate through the functions of the mobile app to ensure competence, especially on the sections involving self-management. Patients will be coached to use the app and to review their progress at home (e.g., self-monitoring of their weight, BP and blood glucose) and recording of their PA and diet. Particular attention will be given to specific pages of the app which summarise patients’ achievement in their self-management goals and clinical outcomes. These achievements will be rewarded with a star rating in accordance with the persuasive technology theory. PCPs and the nurse will discuss self-management progress and goals using the app with the patients at each follow-up visit. The goal of the intervention is to improve patient activation and self-management behaviours through patient–physician collaboration and regular reinforcement to ensure sustainability. Figure 3 illustrates the delivery of the EMPOWER-SUSTAIN Self-Management e-Health Intervention.

### Monitoring the intervention

During the 6-month intervention period, patients are required to use the EMPOWER-SUSTAIN self-management mobile app for a cumulative period of 2 h and be seen at least once by the PCPs for follow-up care. A separate web interface will be created for PCPs to monitor patients’ log-in frequency and duration of use of the tool. Patients who do not comply with the use and follow-up requirements will be considered as lost to follow-up. Patients who are lost to follow-up or who withdraw from the trial will not be replaced. Analysis will be by intention to treat (ITT). There is no other specific concomitant care and intervention that are permitted or prohibited during the trial.

### Outcome measures

Outcome measures are divided into primary and secondary outcomes. These measures will be obtained from both intervention and control groups at baseline and 6 months after the delivery of the intervention.

### Primary outcome

The primary outcome will be measured by the mean change in patient activation score using the Patient Activation Measure short form–Malay version (PAM-13-M) questionnaire [24].

### Secondary outcomes

1. Change in mean WC
2. Change in mean BMI
3. Change in mean systolic and mean diastolic BP
4. Change in PA level will be measured by the International Physical Activity Questionnaire short form–Malay version (IPAQ-M) [70].
5. Change in eating behaviour will be measured by the Dutch Eating Behaviour Questionnaire–Malay version (DEBQ-M) [71].
6. Change in patients’ perceptions and experiences of receiving care for chronic conditions will be measured by the Patient Assessment of Chronic Illness Care–Malay version (PACIC-M) questionnaire [72].
7. Change in patient–physician satisfaction will be measured by the Skala Kepuasan Interaksi Perubatan (SKIP-11) questionnaire [73].
8. Change in accuracy of CVD risk perception will be measured by the absolute difference between the perceived absolute 10-year CV risk and the actual calculated risk by Framingham Risk Score (FRS) general CVD risk prediction chart [74–76].

### Control group

**Fig. 4:**
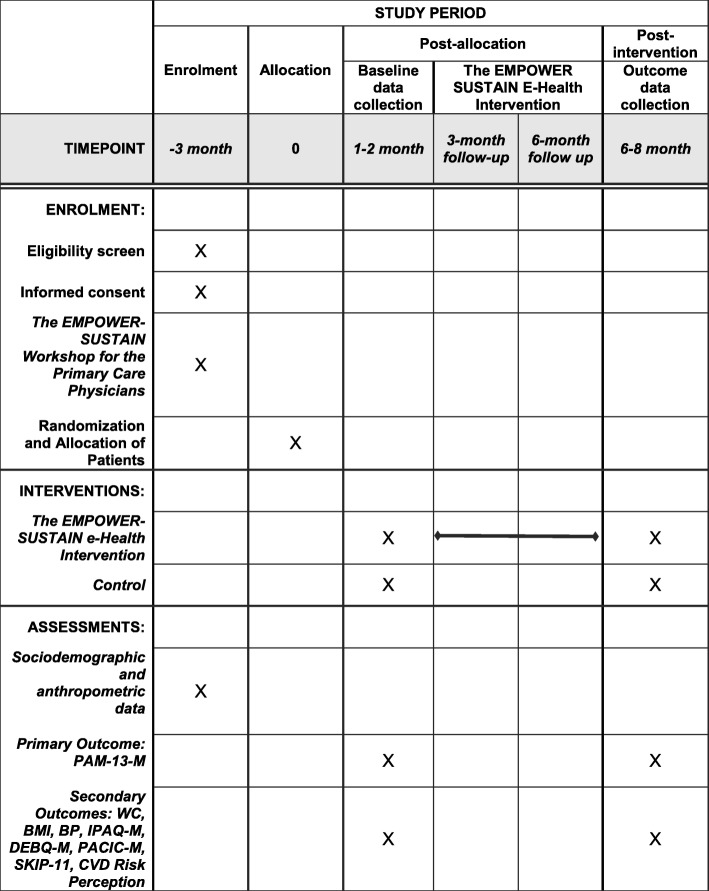
The EMPOWER-SUSTAIN Standard Protocol Items: Recommendations for Interventional Trials (SPIRIT) schedule of enrolment, intervention and assessment

The control group will continue to receive usual care at the university primary care clinic. They will be given the EMPOWER-SUSTAIN Global CV Risks Self-Management Booklet, as this is considered as usual care at the university primary care clinic. The EMPOWER-SUSTAIN self-management mobile app will be made available to the control group at the end of the study. During the course of the study, there will be no limit to the number of clinic visits that a patient is allowed to make in either the intervention or control group. Figure 4 shows the EMPOWER-SUSTAIN schedule of enrolment, interventions and assessments according to SPIRIT guidelines [40].

### Study procedures

All interviewers and investigators will be trained regarding the study procedures prior to the conduct of the study to minimise variability in the method of data collection. Data will be collected at baseline and 6 months after delivery of the intervention. The study procedures will be conducted in accordance with the Malaysian Guideline for Good Clinical Practice, Fourth Edition, 2018 [77].

### Demographic and anthropometric data collection

A standardised case report form (CRF) will be used to collect socio-demographic information of the patients (age, sex, ethnicity, patient contact details, educational attainment, and occupation), smoking status (including the number of cigarettes smoked per day for current smokers) and other clinical information (presence of comorbidities, past medical history and family history). Data on pharmacological treatment will be systematically collected from the medical records of the study patients using CRFs at baseline and at 6-month follow-up in both the intervention and control clinics.

Height and weight will be measured using the Seca 769 Digital Medical Scale stadiometer (Seca, Hamburg, Germany). Weight will be measured with the patient in light clothing, without shoes on the scale, and with a precision of 0.1 kg. Height will be measured to 0.1 cm using the stretch stature method of the stadiometer and then converted to metres. BMI will be calculated using the standard formula (weight in kg)/(height in metres)^2^.

WC will be measured to the nearest 0.1 cm using non-stretchable measuring tape with the patient standing in a relaxed position and arms at the side. The measurement will be taken at the midpoint between the lower rib margin (12th rib) and the top of the iliac crest in horizontal plane. Each measurement will be repeated twice. If the measurements are within 1 cm of one another, the average will be calculated. If the difference between the two measurements exceeds 1 cm, both measurements will be repeated.

Patients will be made to rest and will not be allowed to eat, drink caffeinated beverages, exercise, smoke cigarettes, or climb stairs for at least 15 min before BP measurements are taken. BP will be measured twice, 2 min apart, on the right arm in a sitting position, using an Omron IA2 model automatic digital blood pressure monitor (Omron Healthcare, Kyoto, Japan). Each patient will be seated upright with his/her right arm supported at the heart level. The mean of the first and second systolic and diastolic measurements will be reported as the BP value for individual patients if the difference is within 5 mmHg. If the readings differ by more than 5 mmHg, the measurements will be repeated.

### Administration of the questionnaires

Patients in both the intervention and control arms will be given a set of questionnaires to be self-administered, which include PAM-13-M, IPAQ-M, DEBQ-M, PACIC-M, SKIP-11 and a visual analogue scale to record the perceived absolute 10-year CVD risk.

Clear written and verbal instructions will be given on how to complete the questionnaires. Patients will be requested to circle or tick which options suit them the most. Patients will be encouraged to seek clarification from the investigators at any time, should any queries arise. They will also be reminded to answer the questionnaires themselves rather than getting help from their accompanying family members.

Patients will be given a pen to complete the questionnaires at a corner of the clinic equipped with tables and chairs. The investigators will ensure that patients do not interact with each other whilst answering the questionnaires. On average, patients will be expected to take approximately 30 min to complete the set of questionnaires. Once they have finished, they will hand the questionnaires to the investigators, who will then check the responses for completeness.

### Study tools

#### PAM-13-M questionnaire

The PAM-13 consists of 13 items measuring patients’ self-reported knowledge, skills and confidence for self-management [24]. It was developed in the English language using a Rasch model [24]. Each item has five response categories with scores ranging from 1 to 5: (1) ‘strongly disagree’, (2) ‘disagree’, (3) ‘agree’, (4) ‘strongly agree’ and (5) ‘not applicable’. The instrument design reflects the four stages of activation in a progressing degree of difficulty of the items: level 1 (patients believe that their role is important: items 1 and 2), level 2 (patients have confidence and knowledge to take action: items 3–8), level 3 (taking action: items 9–11) and level 4 (staying on course under stress: items 12 and 13). According to the PAM-13 scoring guidelines, the raw scores are transformed through natural logarithm to achieve a better expression of the relative distance between the scores. Then, items are transformed to a standardised metric ranging from 0 to 100 (0 = lowest activation; 100 = highest activation). The score is calculated by summing the raw scores and mapping the sum onto a scale of 0–100. A higher score of PAM-13 indicates a high level of patient activation [24]. The PAM-13 is one of the most extensively used, widely translated and tested instruments worldwide in measuring patient activation level in self-management. It has been translated into the Malay language and is currently being validated in the study population. Research licence to use this questionnaire will be obtained from Insignia Health, University of Oregon.

#### IPAQ-M

The IPAQ was developed to measure health-related PA in the English language [78] and was previously translated into the Malay language and validated in the Malaysian population [70]. The IPAQ-M short version comprises of 12 items, covering vigorous, moderate, walking, sitting and sleeping activities [70]. Patients are required to report the activities performed during the last 7 days and to include only activities that lasted 10 min or more per session. IPAQ will be scored according to its scoring protocol [79]. Continuous score will be expressed as metabolic equivalent task (MET) minutes per week: MET level × minutes of activity/day × days per week [79]. The scores will then be categorised into ‘low’, ‘moderate’ and ‘vigorous’ PA levels in accordance with the Malaysian CPG on Primary and Secondary Prevention of Cardiovascular Disease 2017 [64]. The IPAQ-M short version has acceptable validity for moderate, vigorous and total PA and was found to be reliable for assessing PA of Malay adults [70].

#### DEBQ-M

The DEBQ was developed in the English language [80] and has been translated into the Malay language and validated in the Malaysian population [71]. The DEBQ-M contains 33 items to measure emotional, external and restrained eating behaviours. Emotional eating is assessed by 13 items, whereas external and restrained eating behaviours are assessed by 10 items each. The questions that assess the three different behaviours appear in random order in the questionnaire and are answered according to a Likert scale with a scoring system identified as follows: 1 = never, 2 = rarely, 3 = sometimes, 4 = often, and 5 = very often. There are three subscales in the instrument. For each subscale, the score is added and divided by the number of items in the subscale to obtain the average score for emotional, external and restrained eating for a person [71].

#### PACIC-M questionnaire

The PACIC questionnaire consists of a 20-item patient self-report instrument developed in the English language to assess the extent to which patients with chronic disease receive care that aligns with the CCM [81]. It has recently been translated into the Malay language and cross-culturally adapted and validated to produce the PACIC-M [72]. It measures care that is patient-centred, proactive and planned, which includes collaborative goal setting, problem-solving and follow-up support [81]. Each item is scored on a 5-point Likert scale, with 1 being ‘no’ or ‘never’ and 5 being ‘yes’ or ‘always’. The higher the score, the more aligned is the perceived care to CCM. PACIC-M was found to be highly reliable (Cronbach’s α of 0.94 with mean interitem correlation of 0.45) and valid to be used in assessing three CCM model domains [72].

#### SKIP-11 questionnaire

SKIP-11 is the translated and validated Malay version [73] of the Medical Interview Satisfaction Scale [82]. SKIP-11 is used to measure patient–physician interaction satisfaction and consists of 11 questions representing three subdomains of patient–physician interaction satisfaction. There are four questions pertaining to information provision (‘Distress relief’ subdomain), four questions regarding the physician’s communication skills (‘Rapport’ subdomain) and three questions assessing the adherence intent as an outcome of the overall interaction experience (‘Interaction outcome’ subdomain). All 11 items are scored using a 5-point Likert scale whereby for positively worded items, a score of 5 is for ‘strongly agree’ and a score of 1 is for ‘strongly disagree’. For the negatively worded items, score 1 is for ‘strongly agree’ and score ‘5’ is for strongly disagree. Each response will be added together to give a total score within the range of 11 (minimum) and 55 (maximum). Total score for each subdomain is also calculated and analysed, where the minimum and maximum scores are determined by the number of items present in each subdomain. The levels of satisfaction will be determined by the proximity of the score to either the minimum or maximum score for each subdomain. The closer proximity of the score to the maximum score will reflect good satisfaction level and vice versa [73].

#### Accuracy of the perceived absolute 10-year CVD risk

The perceived absolute 10-year risk of heart attack and stroke will be estimated separately by the patients along a visual analogue scale [74]. The average of these values will be taken as the perceived absolute 10-year CVD risk for the patients. The patients’ actual absolute 10-year CVD risk will be calculated using the FRS general CVD risk prediction chart [75, 76], which has been validated in the Malaysian population [83]. The accuracy of the CVD risk perception will be defined as the absolute difference between the perceived and actual risk, which will be inversely related (i.e., the lower the absolute difference, the more accurate the patients’ CVD risk perception) [74].

### Sample size determination

Sample size is calculated using Power and Sample Size Calculation software version 3.1.2 [84], based on the findings of a randomised controlled trial evaluating the effects of a web-based self-management intervention for adults with chronic conditions on patient activation scores, measured by the PAM-13 questionnaire [48]. In the intervention group, the mean patient activation score at baseline was 65.33, and the mean score after the intervention was 71.30 (mean difference, 5.97 ± 9.70; t57 = 4.683; *P* < 0.001) [48]. In the control group, the mean patient activation score at baseline was 66.89, and the mean score at the end of the study period was 68.93 (mean difference, 2.04 ± 10.01; t67 = 1.677; *P* = 0.10) [48]. Therefore, the mean difference between the two groups was 3.93.

Based on this assumption, a sample size of 97 patients per group is sufficient to detect mean difference of δ = 3.93 in the patient activation score between the two groups, with a standard deviation of σ = 9.70 using a two-tailed *t* test of difference between means with 80% power (power = 0.8), 5% level of significance (α = 0.05), and sample size ratio of 1:1 between the two groups (m = 1). After considering a drop-out rate of 20%, the sample size required is 116 patients per group, giving a total of 232 patients to be recruited for this study.

### Randomisation

Randomisation of patients to either the EMPOWER-SUSTAIN Self-Management e-Health Intervention (I) or usual care (C) will be done using randomised block design by a research assistant. Random allocation will be done in order to keep the sizes of the groups similar. In this study, the block randomisation size will be four times the number of treatment arms (i.e., block size of 2 by 2). With two treatment arms of the EMPOWER-SUSTAIN Self-Management e-Health Intervention (I) or usual care (C), the possible treatment allocations within each block [85] will be as follows: IICC, ICIC, ICCI, CCII, CICI, CIIC. A research assistant will generate the allocation sequence, enrol the participants and assign the patients to the intervention or control arms according the sequence. The generated sequence will be placed in sealed envelopes to ensure that it is concealed from the PCPs until the intervention is assigned. Blinding is not possible, owing to the complexity of the intervention.

### Data management

Each CRF will be given a unique identifier. Data collected using the CRF and all the questionnaires will be checked by a research assistant to ensure completeness. If any missing data are found, the patients will be contacted again via telephone. Double data entry into the IBM SPSS Statistics version 24 software (IBM, Armonk, NY, USA) will be conducted, and data cleaning will be done to manage outliers, missing values, and inconsistencies. The clean datasets for baseline and outcome will be used for analysis. Data will be stored in a secure database at the Institute of Pathology, Laboratory and Forensic Medicine (I-PPerForM), Universiti Teknologi MARA (UiTM).

### Statistical analysis

The analysis will be conducted using IBM SPSS Statistics version 24 software.

#### Descriptive analysis

Frequency distribution, a measure of central tendency and dispersion, will be produced. For the continuous data, it will be presented by mean and standard deviation or median based on the normality of the data. For the categorical data, it will be presented by absolute number and the corresponding percentage.

#### Effectiveness analysis

ITT analysis will be applied to measure the potential effectiveness of the EMPOWER-SUSTAIN Self-Management e-Health Intervention on the primary and secondary outcome measures based on the initial treatment assignment. Mixed model repeated measures analysis of variance will be carried out to evaluate the potential effectiveness (i.e., to compare the mean changes in patient activation, PA, diet, patients’ chronic illness care, and patient–physician interaction satisfaction scores) with regard to perceived absolute 10-year CVD risk within and between the intervention and control groups at baseline and 6-month follow-up.

### Feasibility outcomes

Process evaluation to assess the integrity of the randomised controlled trial protocol will be conducted. These include recruitment rate, methods of randomisation, retention rate, appropriateness of the primary and secondary outcome measures, sample size calculation, whether the intervention could be delivered as intended, and methods of statistical analysis to evaluate the potential effectiveness. Qualitative studies to explore facilitators and barriers in delivering the intervention among PCPs and using the intervention among patients will also be conducted. However, detailed methods for the qualitative studies are beyond the scope of this paper.

### Data monitoring

Data monitoring will be done by the EMPOWER-SUSTAIN investigators. Data on any adverse event or unintended effect of trial intervention or trial conduct will be collected, assessed, reported and managed by the investigators. An external data monitoring committee is not needed, because the intervention does not involve a new pharmacological agent or regulated device.

## Discussion

To the best of our knowledge, the EMPOWER-SUSTAIN project is the first self-management e-health intervention designed for patients with MetS in the Malaysian primary care setting. The pilot randomised controlled trial will be conducted to evaluate the feasibility and potential effectiveness of the EMPOWER-SUSTAIN intervention, including recruitment rate, methods of randomisation, retention rate, selection of primary and secondary outcome measures, sample size calculation, whether the intervention could be delivered as intended, and methods of statistical analysis to evaluate the potential effectiveness [44, 45]. All of these aspects will be useful for further exploration in a larger definitive trial.

The EMPOWER-SUSTAIN Self-Management e-Health Intervention is expected to yield important new evidence on the potential improvements of patient activation and self-management behaviours among patients with MetS in a developing country. It is hypothesised that patients’ activation, WC, BMI, blood pressure, PA level, eating behaviour, perception and experience of receiving chronic disease care, patient–physician satisfaction, and perceived absolute 10-year CVD risk would improve with the EMPOWER-SUSTAIN Self-Management e-Health Intervention.

The EMPOWER-SUSTAIN intervention is a complex, multifaceted chronic disease management strategy based on three CCM elements (i.e., delivery system design, self-management support and decision support). It consists of training the PCPs, nurse, and patients to use the EMPOWER-SUSTAIN web-based self-management mobile app, strengthening patient–physician relationship, and reinforcing the use of relevant CPG for management and prescribing. This intervention is developed on the basis of MRC recommendations, guided by the best available evidence and appropriate framework [46, 47].

It is well established that the CCM is one of the best-known models to transform chronic disease care [11]. The EMPOWER-SUSTAIN intervention focuses on linking informed, activated patients with proactive and prepared practice team (i.e., PCPs and nurse) [11, 86]. The self-management mobile app is developed as a tool for health care providers to support and engage patients so that they are empowered with knowledge, skills and confidence to take independent actions to manage their own health [11, 12].

Development of an e-health intervention to support patients’ self-management requires careful planning and the use of theory-based strategies to increase the probability of effectiveness, programme adoption and implementation [34–37]. Therefore, the EMPOWER-SUSTAIN self-management mobile app is developed using the persuasive technology theory [39]. It consists of evidence-based content presented in a user-friendly interface, with incorporation of interactivity, reward system, problem-solving assistance, patient–physician collaboration, and regular reinforcement to ensure sustainability [34–37, 49, 87–89]. It is designed to aid and motivate people to adopt a positive attitude and behaviour change through persuasion and social influence [39].

To ensure effective patient–physician collaboration, PCPs will be trained prior to the delivery of intervention. In the EMPOWER-SUSTAIN Workshop, PCPs and a nurse will be trained on the basis of SDT, an evidence-based framework of human motivation and personality theory which supports the individual’s experience of autonomy, competence, relatedness and engagement for activities [65]. It is useful for them to understand that individuals’ behaviour change can be influenced by the role of a health care provider [65]. They will also be trained on MI techniques and health-coaching skills to facilitate patients’ autonomous self-regulation to enhance their behaviour change by enabling patients to develop individualised action plans [65–68].

PCPs assisted by a nurse will then train individual patients on how to use the EMPOWER-SUSTAIN self-management mobile app based on the KTA Framework [69]. This framework is chosen because it is context-focused, enables knowledge-producer and knowledge-user collaboration, and emphasises sustainability [69]. The KTA Framework incorporates the need to adapt the knowledge to fit with individual context. This framework is particularly useful for emphasising the collaboration between knowledge producers and knowledge users [69]. Sustainable knowledge use is essential, given the chronic nature of MetS and the associated CV risk factors. Apart from the KTA Framework, the PCPs and nurse will also apply the MI techniques and health-coaching skills to influence behaviour change in these patients. A systematic review on the effectiveness of MI in primary care has reported positive outcomes of this intervention on health behaviour change [50].

Another essential component of the CCM is clinical decision support [11]. Clinical management should be tailored to individual patients’ needs, guided by evidence-based decision support tool (i.e., the CPG) [11]. Therefore, PCPs will be trained to use the relevant evidence-based CPG to support management and prescribing for patients with MetS. This would empower PCPs to improve their clinical management [90].

## Conclusion

Ultimately, the results of this pilot study will determine the feasibility of this multifaceted e-health intervention, as well as indicate more useful aspects of this intervention for further exploration in a larger trial. This study will also provide evidence of potential effectiveness of a multifaceted intervention involving a web-based self-management mobile app, which is developed on the basis of CCM and persuasive technology theory in the primary care setting. It is hoped that the evidence derived from this study will provide a platform to support a larger definitive clinical trial to evaluate the effectiveness of this e-health self-management intervention in Malaysia.

### Trial status

The development of the EMPOWER-SUSTAIN self-management mobile app is completed. However, due to the Covid-19 pandemic, we are unable to start participant screening and recruitment in March 2020 as previously planned. This study is expected to be delayed by 6 months. We hope to commence participant screening and recruitment in September 2020. The expected date of completion of patient recruitment is October 2020. Baseline data collection is planned to start in December 2020. The intervention is planned to be delivered for 6 months from January to June 2021. The outcome data will be collected in July to August 2021. The expected date of completion of this pilot trial is 31 December 2021.

## Supplementary information

**Additional file 1.** The EMPOWER-SUSTAIN SPIRIT checklist.

**Additional file 2.** The EMPOWER-SUSTAIN mobile app mock prototype.

**Additional file 3.** The EMPOWER-SUSTAIN patient consent form.

**Additional file 4.** The EMPOWER-SUSTAIN physician consent form.

## Data Availability

Data will be kept at the Institute of Pathology, Laboratory and Forensic Medicine (I-PPerForM), Universiti Teknologi MARA (UiTM), Sungai Buloh Campus, Jalan Hospital, 47000 Sungai Buloh, Selangor, Malaysia. Data can be shared upon request and are subject to the data protection regulations.

## Abbreviations

ACS: Acute coronary syndrome
BMI: Body mass index
BP: Blood pressure
CCM: Chronic Care Model
CONSORT: Consolidated Standards of Reporting Trials
CPG: Clinical practice guidelines
CRF: Case report form
CV: Cardiovascular
CVD: Cardiovascular disease
DEBQ-M: Dutch Eating Behaviour Questionnaire–Malay version
FBG: Fasting blood glucose
FRS: Framingham Risk Score
HbA1c: Haemoglobin A1c
HDL-c: High-density lipoprotein cholesterol
HPT: Hypertension
IDF: International Diabetes Federation
IPAQ-M: International Physical Activity Questionnaire–Malay version
ITT: Intention to treat
JIS: Joint Interim Statement
KTA: Knowledge to Action Framework
MET: Metabolic equivalent task
MetS: Metabolic syndrome
MI: Motivational interview
MOH: Ministry of Health
MRC: Medical Research Council
PA: Physical activity
PACIC-M: Patient Assessment of Chronic Illness Care–Malay version
PAM-13-M: Patient Activation Measure short form–Malay version
PCP: Primary care physician
SD: Standard deviation
SDT: Self-determination theory
SKIP-11: Skala Kepuasan Interaksi Perubatan-11
SPIRIT: Standard Protocol Items: Recommendations for Interventional Trials
TG: Triglycerides
WC: Waist circumference
