# Supplementary material for: The EMPOWER-SUSTAIN e-Health Intervention to improve patient activation and self-management behaviours among individuals with Metabolic Syndrome in primary care: study protocol for a pilot randomised controlled trial
Source: Trials. 2020 Apr 5;21:311. doi: 10.1186/s13063-020-04237-x (PMC7130454; doi:10.1186/s13063-020-04237-x)
Supplement: Supplementary file 2 — Additional file 2. The EMPOWER-SUSTAIN mobile app mock prototype. [file 13063_2020_4237_MOESM2_ESM.pdf]

LOGIN

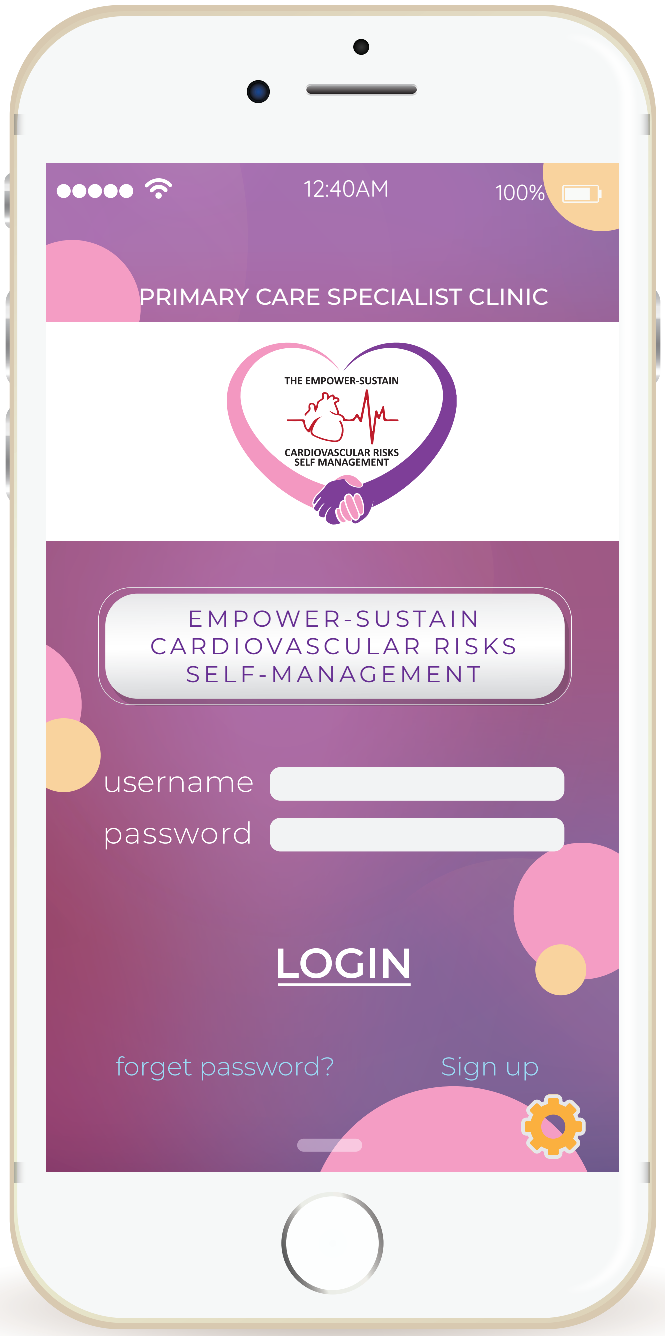

12:40AM
100%
PRIMARY CARE SPECIALIST CLINIC
THE EMPOWER-SUSTAIN
CARDIOVASCULAR RISKS
SELF MANAGEMENT
EMPOWER-SUSTAIN
CARDIOVASCULAR RISKS
SELF-MANAGEMENT
username
password
LOGIN
forget password?
Sign up

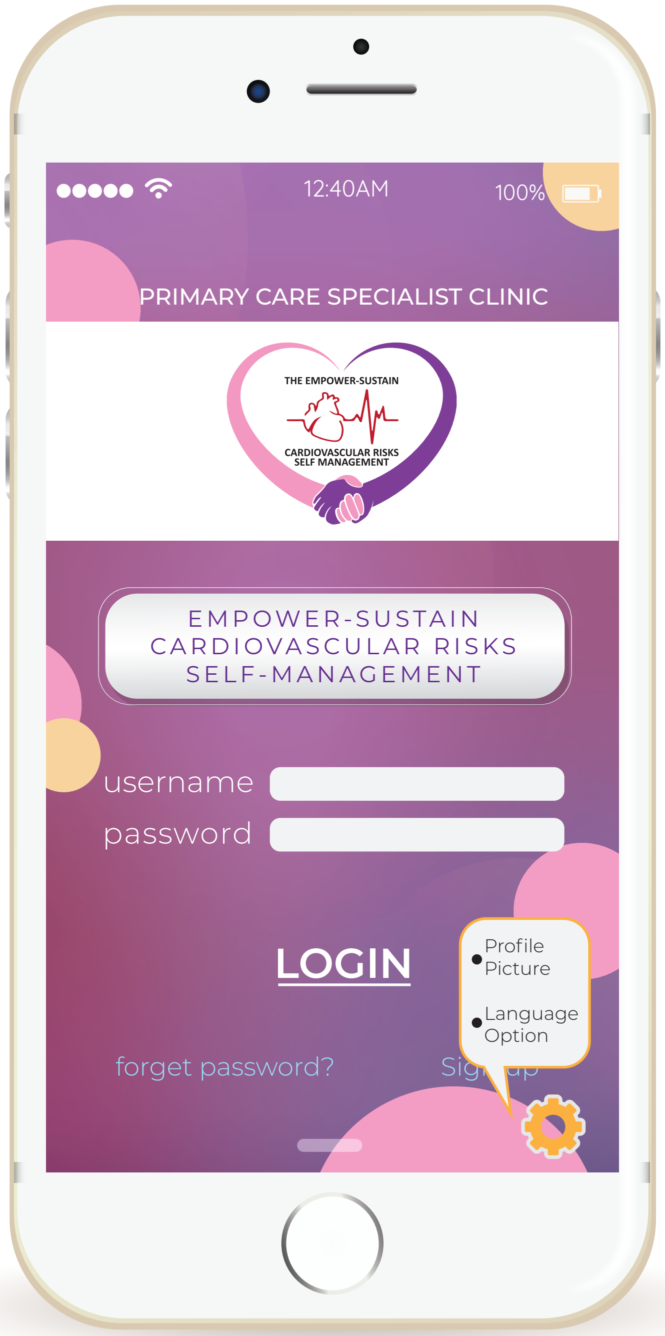

12:40AM
100%
PRIMARY CARE SPECIALIST CLINIC
THE EMPOWER-SUSTAIN
CARDIOVASCULAR RISKS
SELF MANAGEMENT
EMPOWER-SUSTAIN
CARDIOVASCULAR RISKS
SELF-MANAGEMENT
username
password
LOGIN
Profile Picture
Language Option
forget password?

HOMEPAGE

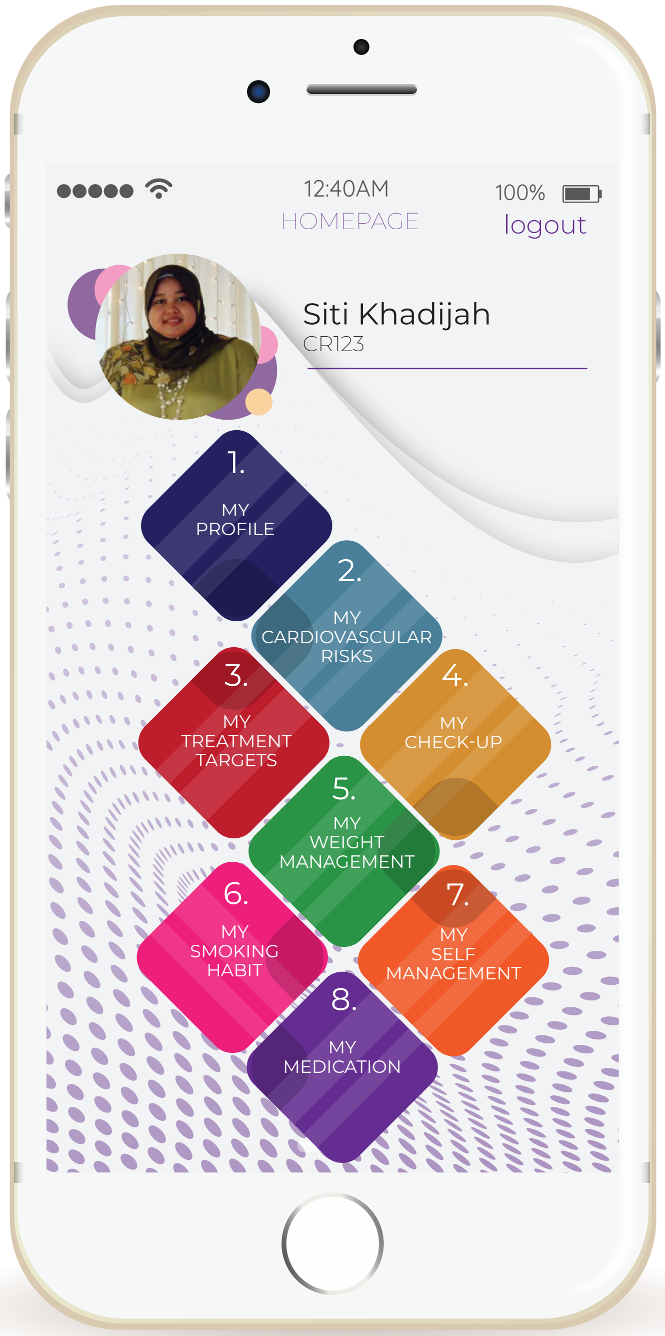

MY PROFILE

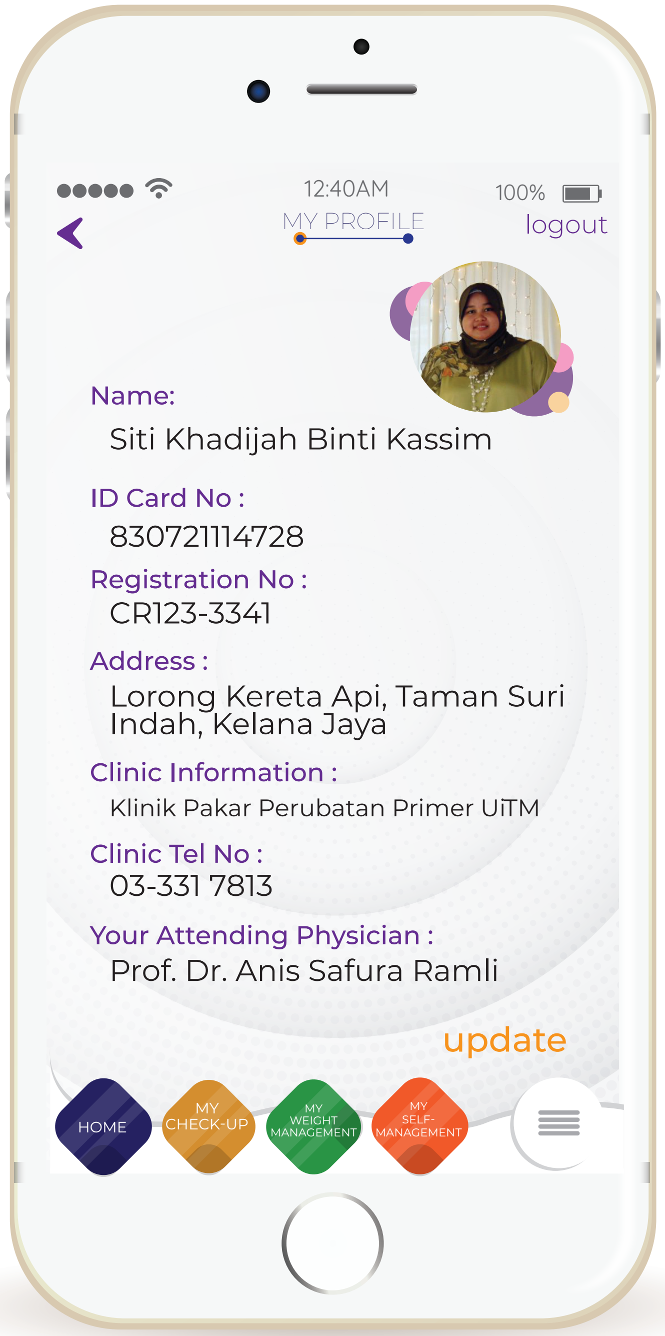

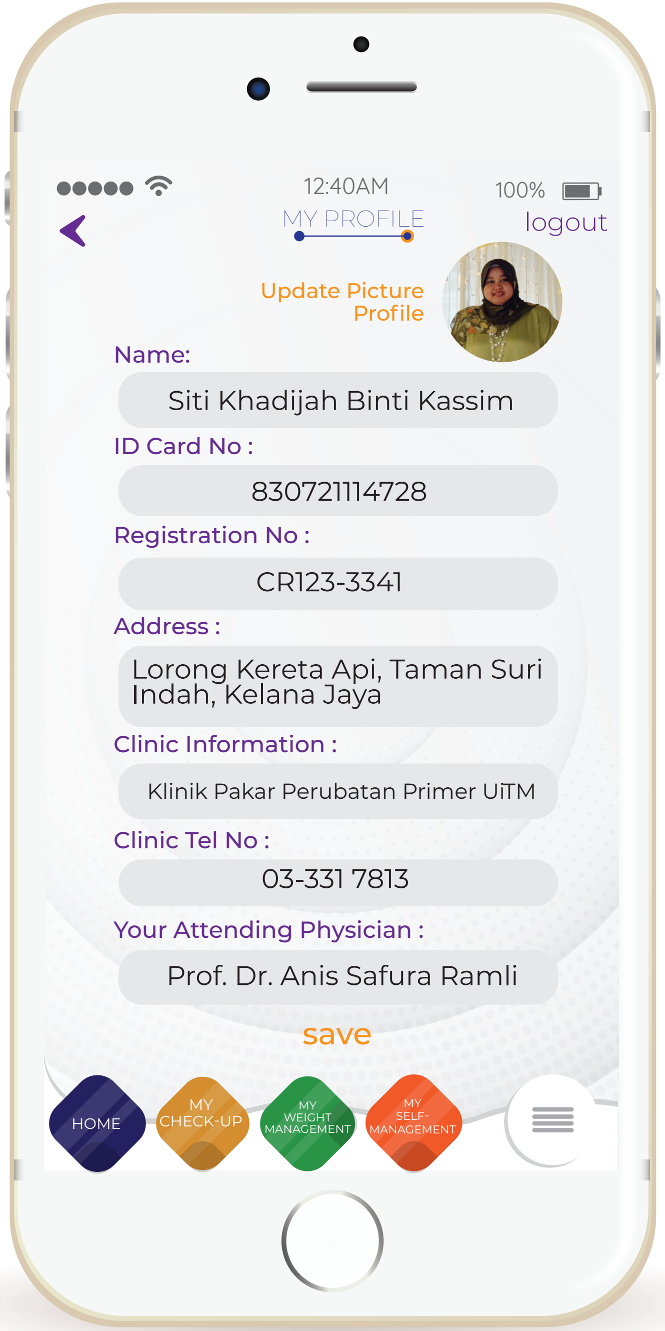

MY CARDIOVASCULAR RISKS

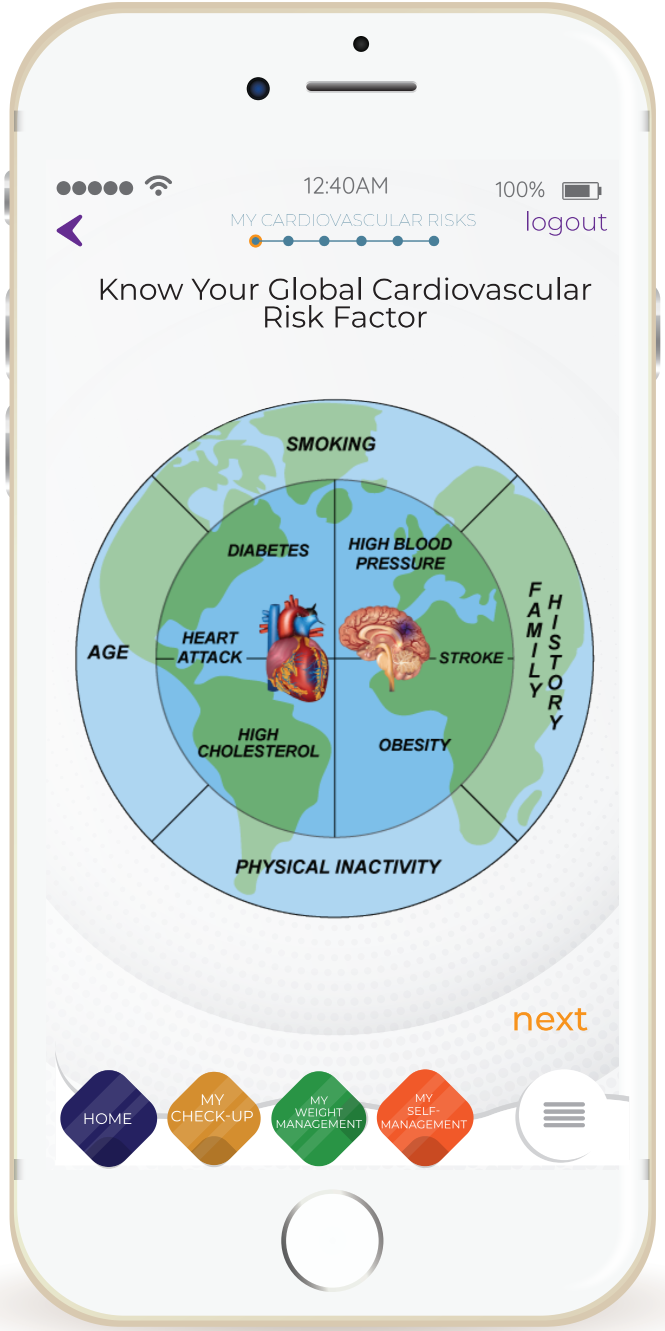

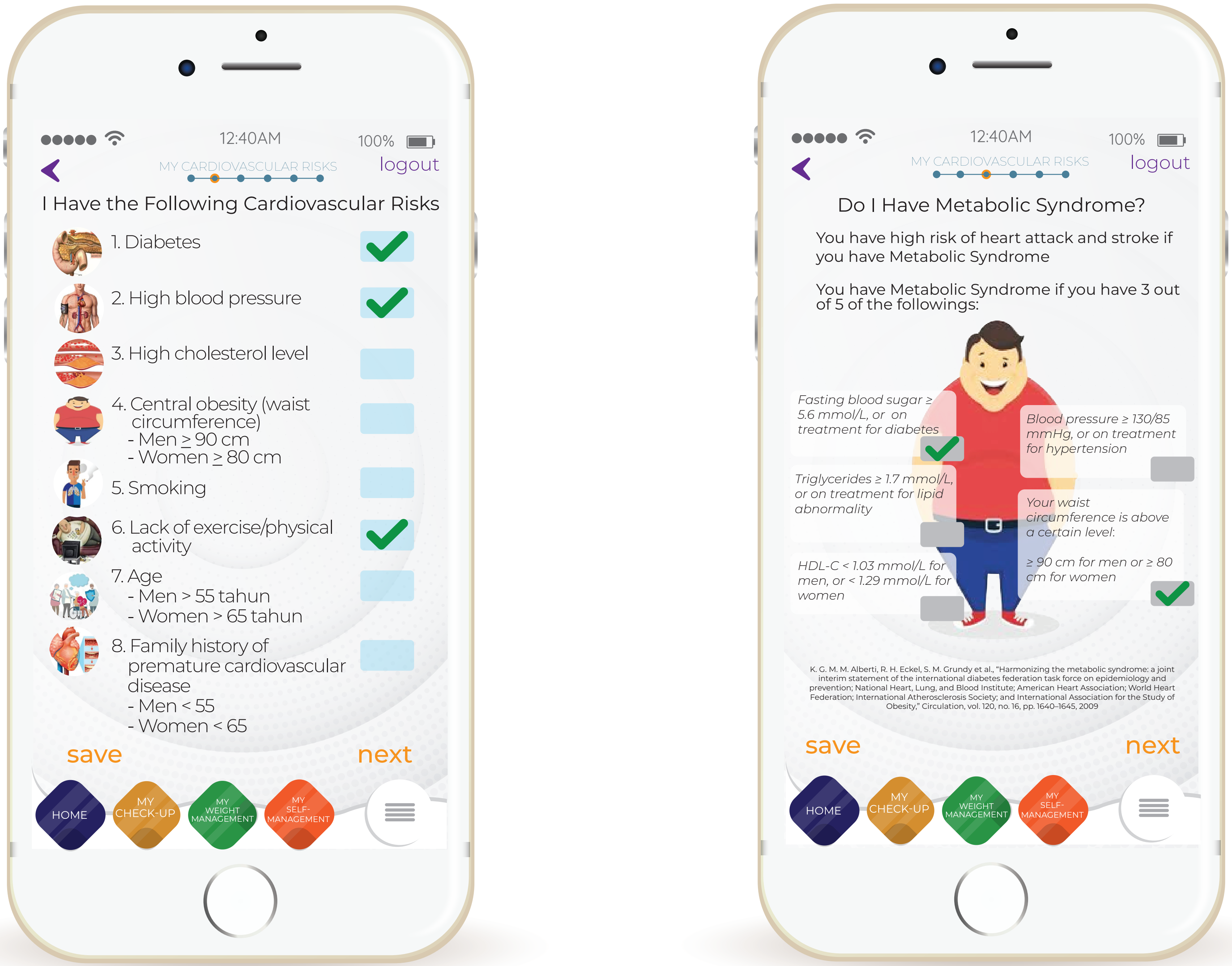
12:40AM
100%
MY CARDIOVASCULAR RISKS
logout
I Have the Following Cardiovascular Risks
1. Diabetes
2. High blood pressure
3. High cholesterol level
4. Central obesity (waist circumference)
- Men ≥ 90 cm
- Women ≥ 80 cm
5. Smoking
6. Lack of exercise/physical activity
7. Age
- Men > 55 tahun
- Women > 65 tahun
8. Family history of premature cardiovascular disease
- Men < 55
- Women < 65
save
next
HOME
MY CHECK-UP
MY WEIGHT MANAGEMENT
MY SELF-MANAGEMENT
12:40AM
100%
MY CARDIOVASCULAR RISKS
logout
Do I Have Metabolic Syndrome?
You have high risk of heart attack and stroke if you have Metabolic Syndrome
You have Metabolic Syndrome if you have 3 out of 5 of the followings:
Fasting blood sugar ≥ 5.6 mmol/L, or on treatment for diabetes
Blood pressure ≥ 130/85 mmHg, or on treatment for hypertension
Triglycerides ≥ 1.7 mmol/L, or on treatment for lipid abnormality
Your waist circumference is above a certain level:
≥ 90 cm for men or ≥ 80 cm for women
HDL-C < 1.03 mmol/L for men, or < 1.29 mmol/L for women
K. G. M. M. Alberti, R. H. Eckel, S. M. Grundy et al., "Harmonizing the metabolic syndrome: a joint interim statement of the international diabetes federation task force on epidemiology and prevention; National Heart, Lung, and Blood Institute; American Heart Association; World Heart Federation; International Atherosclerosis Society; and International Association for the Study of Obesity," Circulation, vol. 120, no. 16, pp. 1640–1645, 2009
save
next
HOME
MY CHECK-UP
MY WEIGHT MANAGEMENT
MY SELF-MANAGEMENT

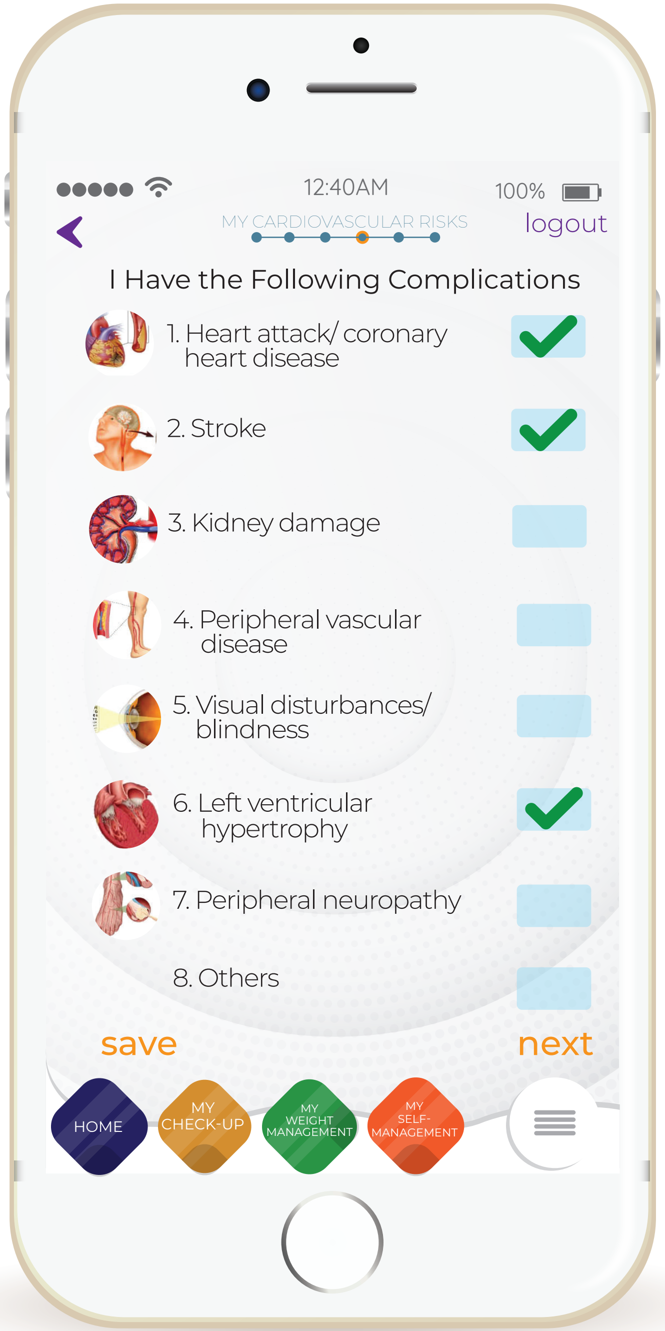
12:40AM
100%
MY CARDIOVASCULAR RISKS
logout
I Have the Following Complications
1. Heart attack/ coronary heart disease
2. Stroke
3. Kidney damage
4. Peripheral vascular disease
5. Visual disturbances/ blindness
6. Left ventricular hypertrophy
7. Peripheral neuropathy
8. Others
save
next
HOME
MY CHECK-UP
MY WEIGHT MANAGEMENT
MY SELF-MANAGEMENT

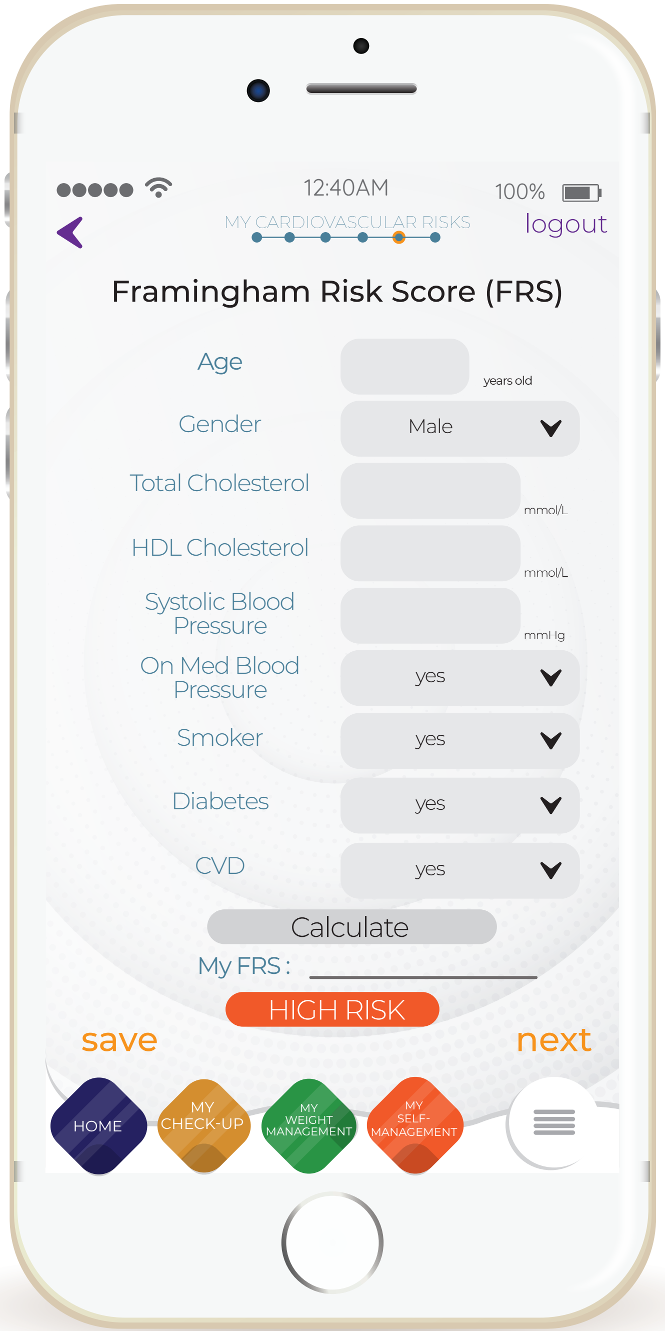
12:40AM
100%
MY CARDIOVASCULAR RISKS
logout
Framingham Risk Score (FRS)
Age
years old
Gender
Male
Total Cholesterol
mmol/L
HDL Cholesterol
mmol/L
Systolic Blood Pressure
mmHg
On Med Blood Pressure
yes
Smoker
yes
Diabetes
yes
CVD
yes
Calculate
My FRS :
HIGH RISK
save
next
HOME
MY CHECK-UP
MY WEIGHT MANAGEMENT
MY SELF-MANAGEMENT

MY TREATMENT TARGETS

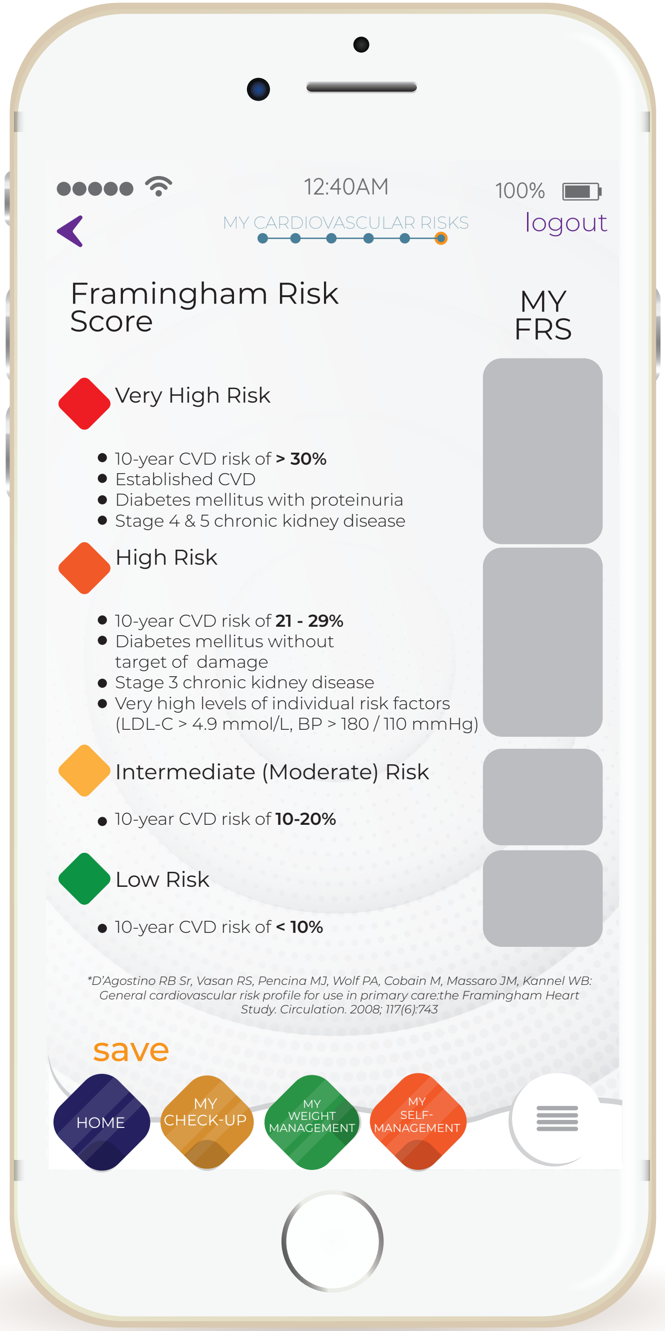

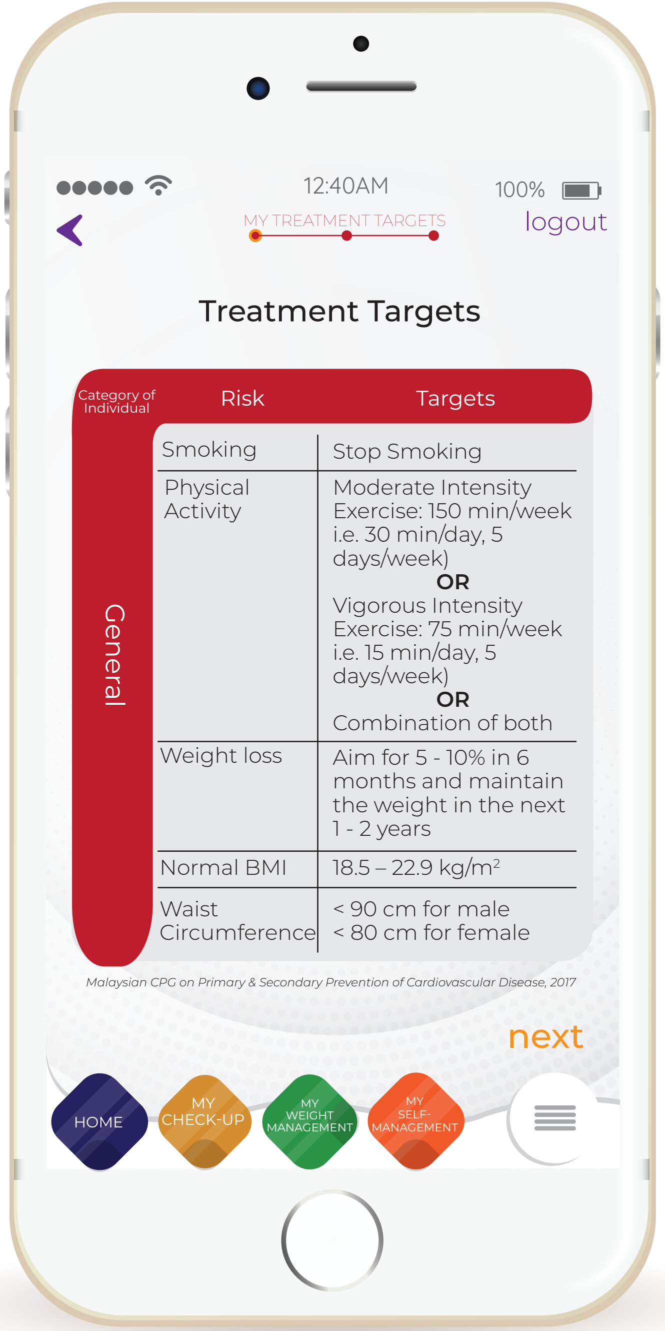

12:40AM 100%

MY TREATMENT TARGETS

logout

Treatment Targets

Category of Individual	Risk	Targets
Without Diabetes	Dyslipidemia	Very High Risk: ◆ LDL-C goal: < 1.8 mmol/L<br><br>High Risk: ◆ LDL-C goal: < 2.6 mmol/L<br><br>Intermediate (Moderate) ◆ and Low Risk: ◆ LDL-C goal: < 3.0 mmol/L
	Blood Pressure	< 140/90 mmHg in most individuals < 80 years of age<br>< 150/90 mmHg in individuals > 80 years of age

Malaysian CPG on Primary & Secondary Prevention of Cardiovascular Disease, 2017

next

HOME | MY CHECK-UP | MY WEIGHT MANAGEMENT | MY SELF-MANAGEMENT

12:40AM 100%

MY TREATMENT TARGETS

logout

Treatment Targets

Category of Individual	Risk	Targets
Diabetes	Pre-prandial or fasting blood sugar	4.4 – 7.0 mmol/L
	Post prandial blood sugar (90-120 mins after a meal)	4.4 – 8.5 mmol/L
	HbA1c	≤ 6.5%
	Blood Pressure	≤ 135/75 mmHg
	LDL-C	≤ 2.6 mmol/L<br>< 1.8 mmol/l in individual with CVD
	HDL-C	> 1.0 mmol/L (male)<br>> 1.2 mmo/L (female)
	Triglycerides	≤ 1.7 mmol/L

Malaysian CPG on Primary & Secondary Prevention of Cardiovascular Disease, 2017

next

HOME | MY CHECK-UP | MY WEIGHT MANAGEMENT | MY SELF-MANAGEMENT

MY CHECK-UP

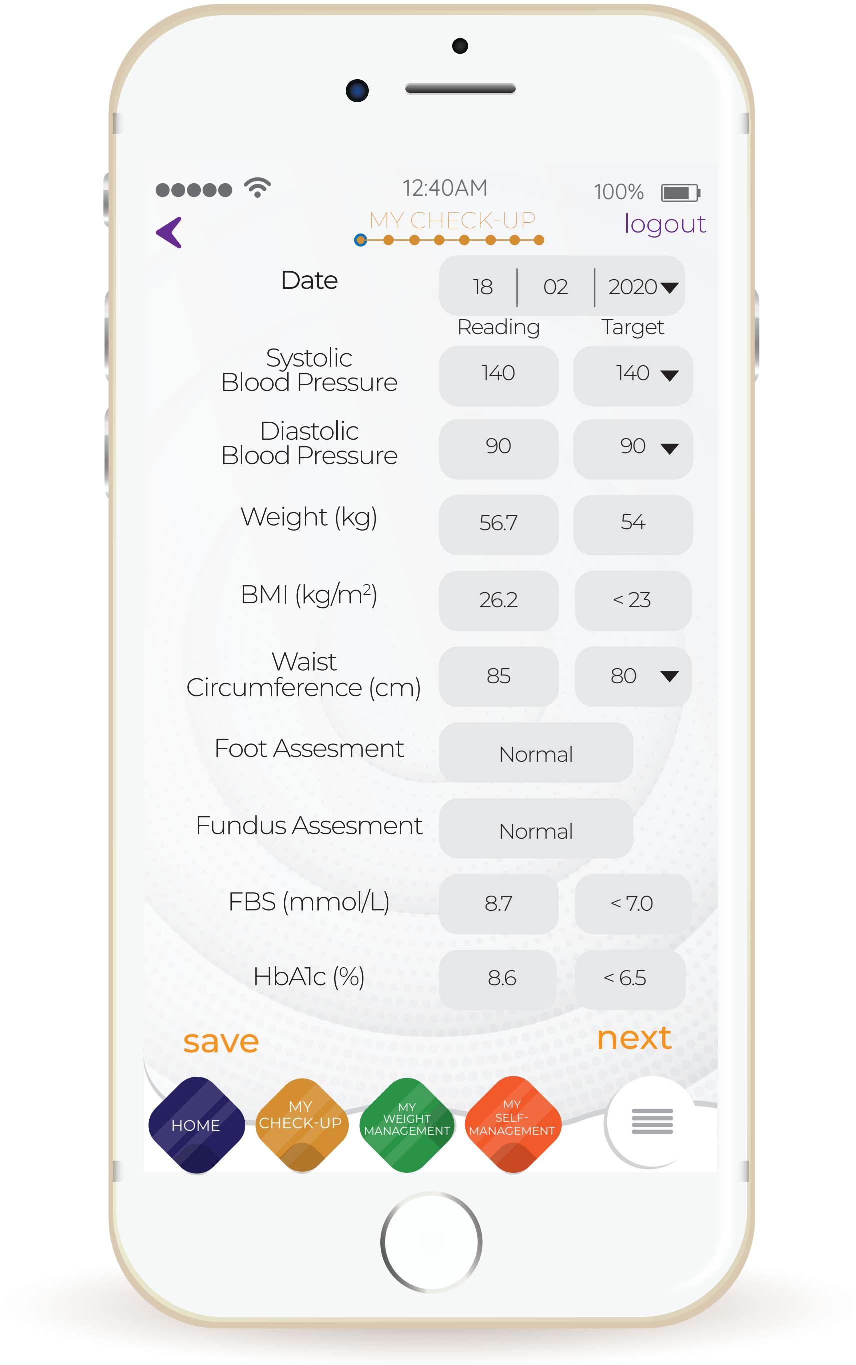
12:40AM
100%
MY CHECK-UP
logout
Date
18
02
2020
Reading
Target
Systolic Blood Pressure
140
140
Diastolic Blood Pressure
90
90
Weight (kg)
56.7
54
BMI (kg/m²)
26.2
< 23
Waist Circumference (cm)
85
80
Foot Assesment
Normal
Fundus Assesment
Normal
FBS (mmol/L)
8.7
< 7.0
HbA1c (%)
8.6
< 6.5
save
next
HOME
MY CHECK-UP
MY WEIGHT MANAGEMENT
MY SELF-MANAGEMENT

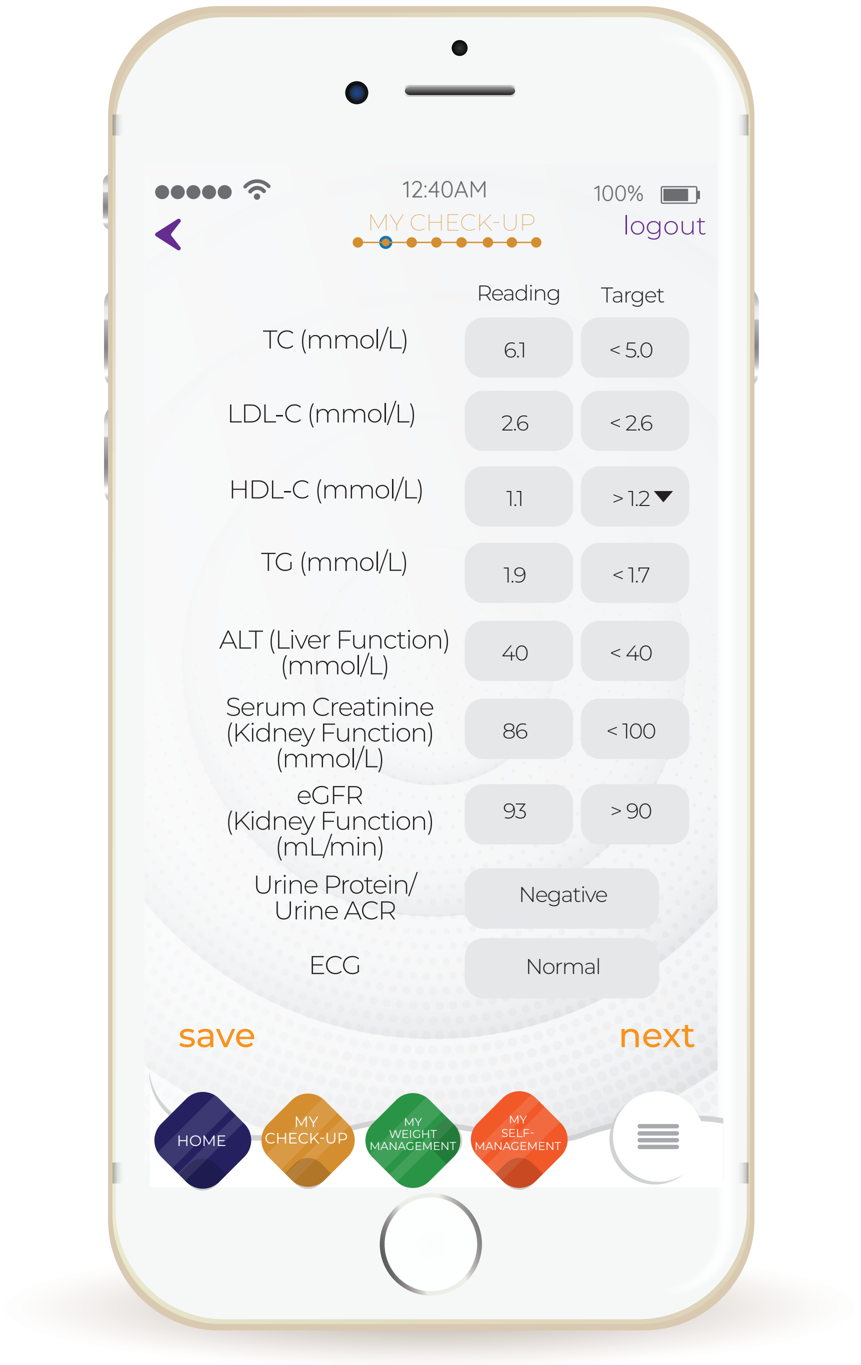
12:40AM
100%
MY CHECK-UP
logout
Reading
Target
TC (mmol/L)
6.1
< 5.0
LDL-C (mmol/L)
2.6
< 2.6
HDL-C (mmol/L)
1.1
> 1.2
TG (mmol/L)
1.9
< 1.7
ALT (Liver Function) (mmol/L)
40
< 40
Serum Creatinine (Kidney Function) (mmol/L)
86
< 100
eGFR (Kidney Function) (mL/min)
93
> 90
Urine Protein/ Urine ACR
Negative
ECG
Normal
save
next
HOME
MY CHECK-UP
MY WEIGHT MANAGEMENT
MY SELF-MANAGEMENT

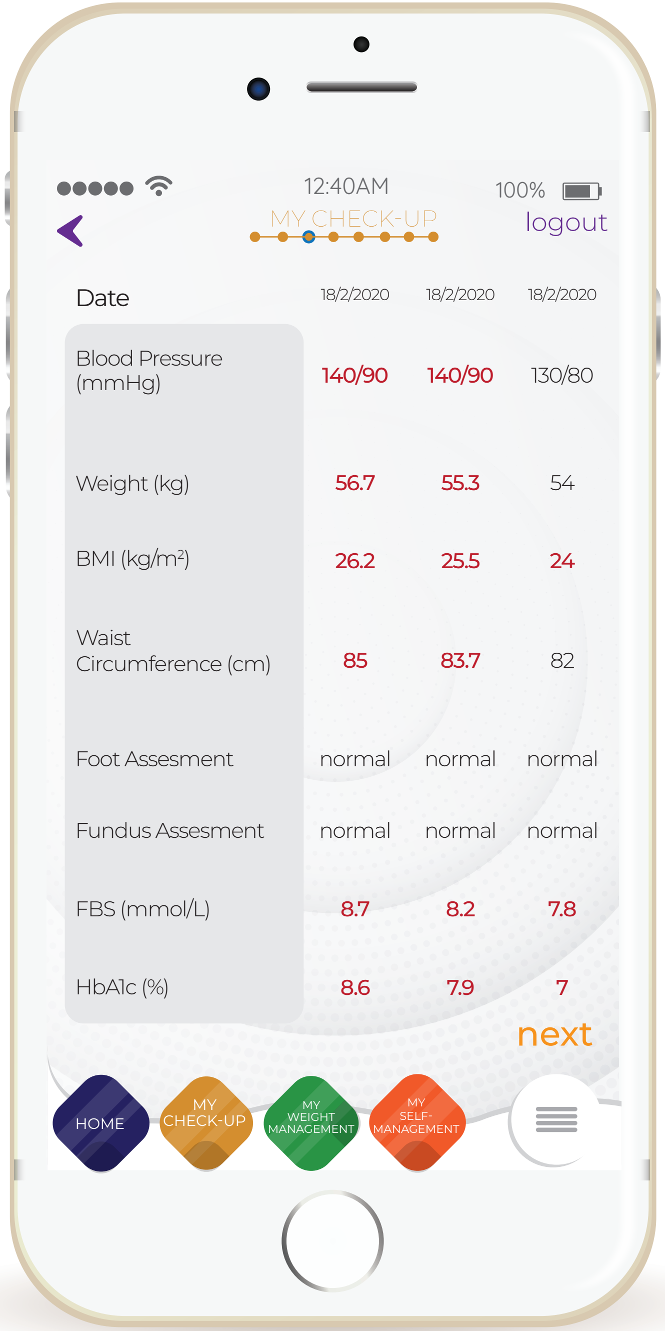
12:40AM
100%
MY CHECK-UP
logout
Date
18/2/2020
18/2/2020
18/2/2020
Blood Pressure (mmHg)
140/90
140/90
130/80
Weight (kg)
56.7
55.3
54
BMI (kg/m²)
26.2
25.5
24
Waist Circumference (cm)
85
83.7
82
Foot Assesment
normal
normal
normal
Fundus Assesment
normal
normal
normal
FBS (mmol/L)
8.7
8.2
7.8
HbA1c (%)
8.6
7.9
7
next
HOME
MY CHECK-UP
MY WEIGHT MANAGEMENT
MY SELF-MANAGEMENT

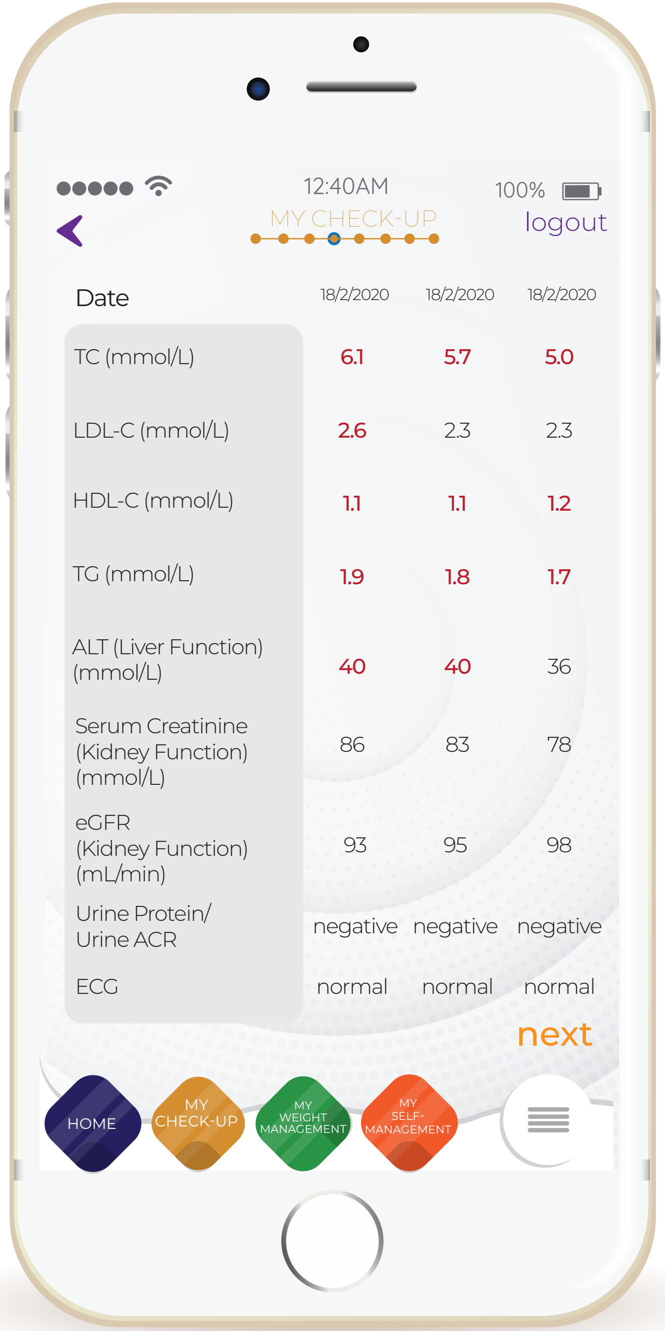
12:40AM
100%
MY CHECK-UP
logout
Date
18/2/2020
18/2/2020
18/2/2020
TC (mmol/L)
6.1
5.7
5.0
LDL-C (mmol/L)
2.6
2.3
2.3
HDL-C (mmol/L)
1.1
1.1
1.2
TG (mmol/L)
1.9
1.8
1.7
ALT (Liver Function) (mmol/L)
40
40
36
Serum Creatinine (Kidney Function) (mmol/L)
86
83
78
eGFR (Kidney Function) (mL/min)
93
95
98
Urine Protein/ Urine ACR
negative
negative
negative
ECG
normal
normal
normal
next
HOME
MY CHECK-UP
MY WEIGHT MANAGEMENT
MY SELF-MANAGEMENT

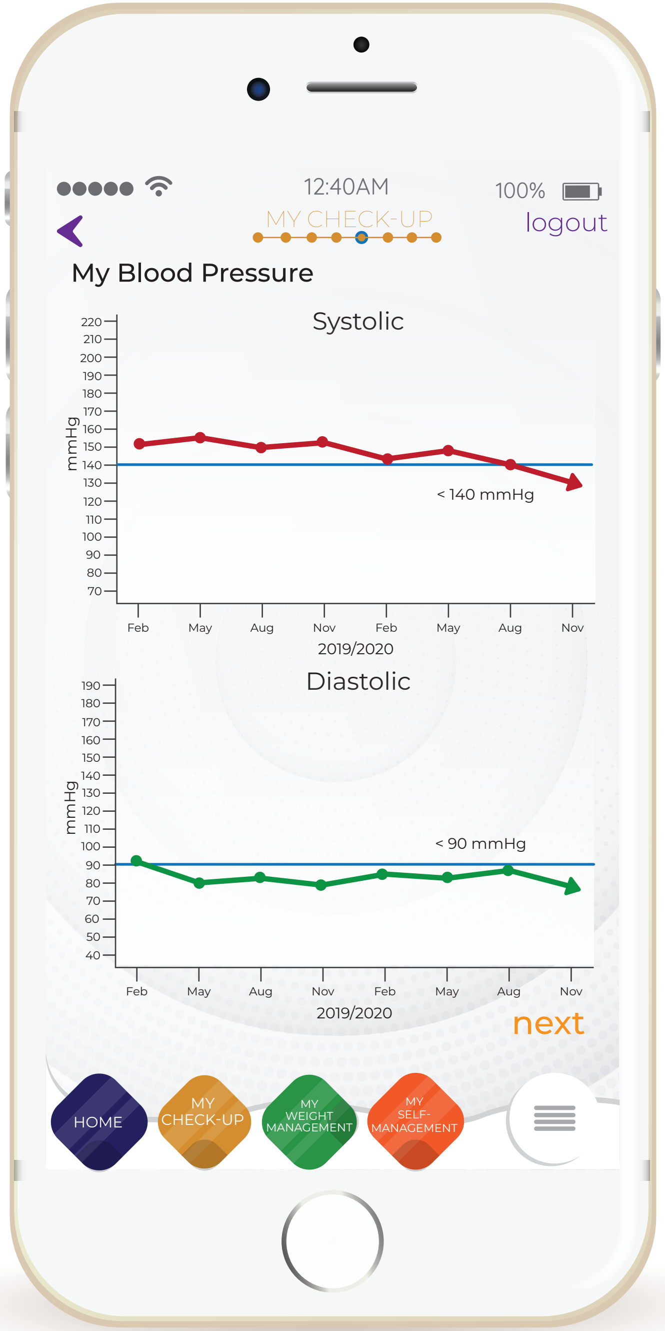

12:40AM
100%
MY CHECK-UP
logout
My Blood Pressure
Systolic
mmHg
220
210
200
190
180
170
160
150
140
130
120
110
100
90
80
70
< 140 mmHg
Feb
May
Aug
Nov
Feb
May
Aug
Nov
2019/2020
Diastolic
mmHg
190
180
170
160
150
140
130
120
110
100
90
80
70
60
50
40
< 90 mmHg
Feb
May
Aug
Nov
Feb
May
Aug
Nov
2019/2020
next
HOME
MY CHECK-UP
MY WEIGHT MANAGEMENT
MY SELF-MANAGEMENT

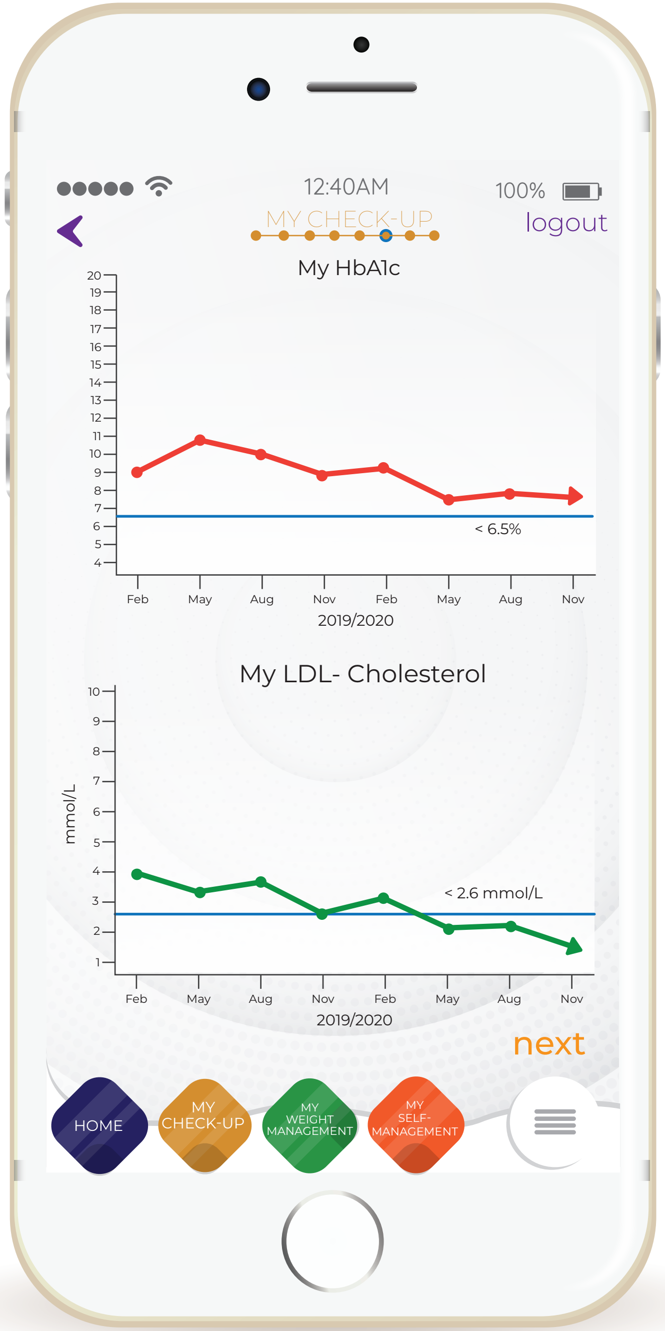

12:40AM
100%
MY CHECK-UP
logout
My HbA1c
20
19
18
17
16
15
14
13
12
11
10
9
8
7
6
5
4
< 6.5%
Feb
May
Aug
Nov
Feb
May
Aug
Nov
2019/2020
My LDL- Cholesterol
mmol/L
10
9
8
7
6
5
4
3
2
1
< 2.6 mmol/L
Feb
May
Aug
Nov
Feb
May
Aug
Nov
2019/2020
next
HOME
MY CHECK-UP
MY WEIGHT MANAGEMENT
MY SELF-MANAGEMENT

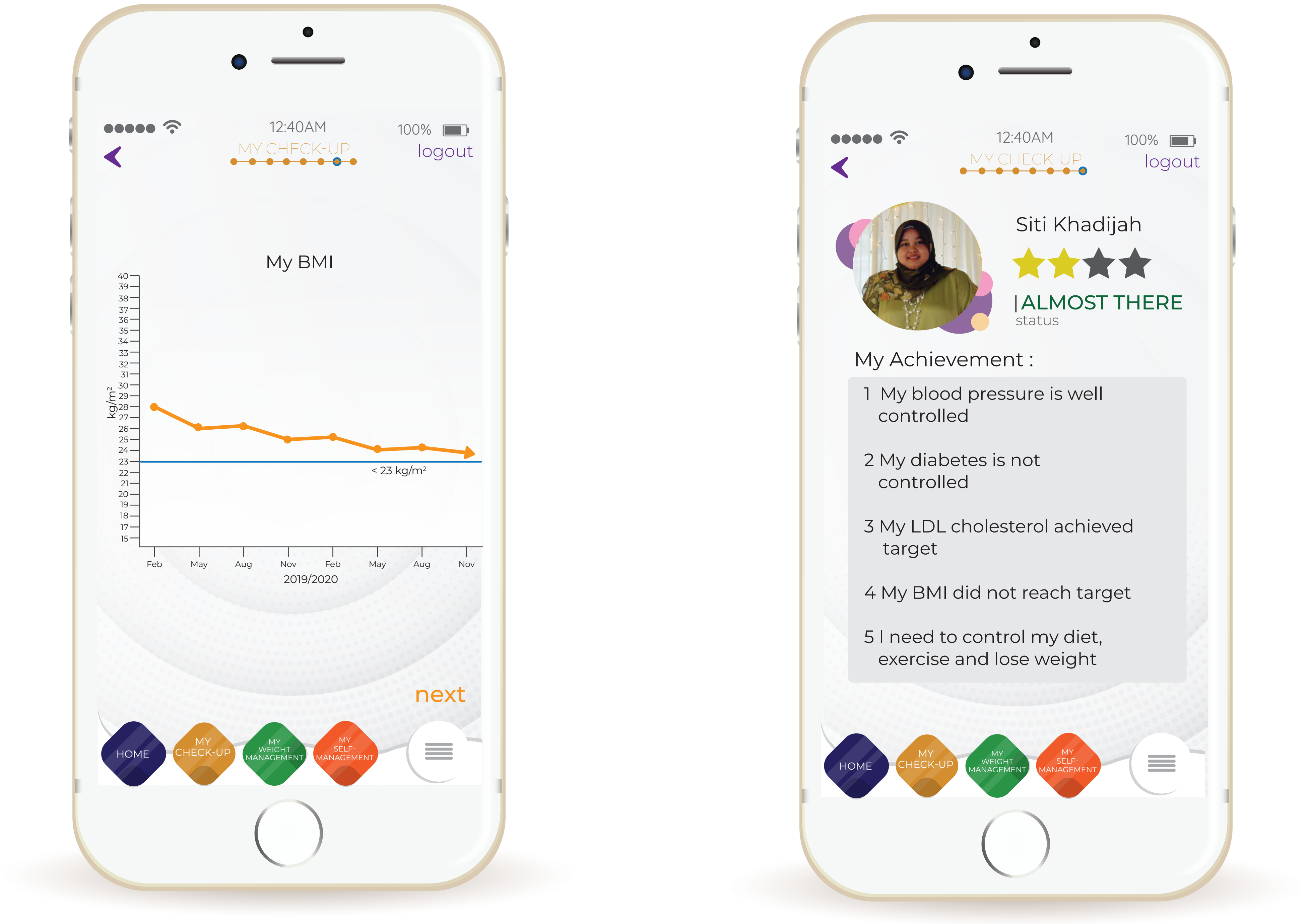
12:40AM
MY CHECK-UP
100%
logout
My BMI
kg/m²
< 23 kg/m²
Feb May Aug Nov Feb May Aug Nov
2019/2020
next
HOME
MY CHECK-UP
MY WEIGHT MANAGEMENT
MY SELF-MANAGEMENT
12:40AM
MY CHECK-UP
100%
logout
Siti Khadijah
| ALMOST THERE
status
My Achievement :
1 My blood pressure is well controlled
2 My diabetes is not controlled
3 My LDL cholesterol achieved target
4 My BMI did not reach target
5 I need to control my diet, exercise and lose weight
HOME
MY CHECK-UP
MY WEIGHT MANAGEMENT
MY SELF-MANAGEMENT

MY WEIGHT MANAGEMENT

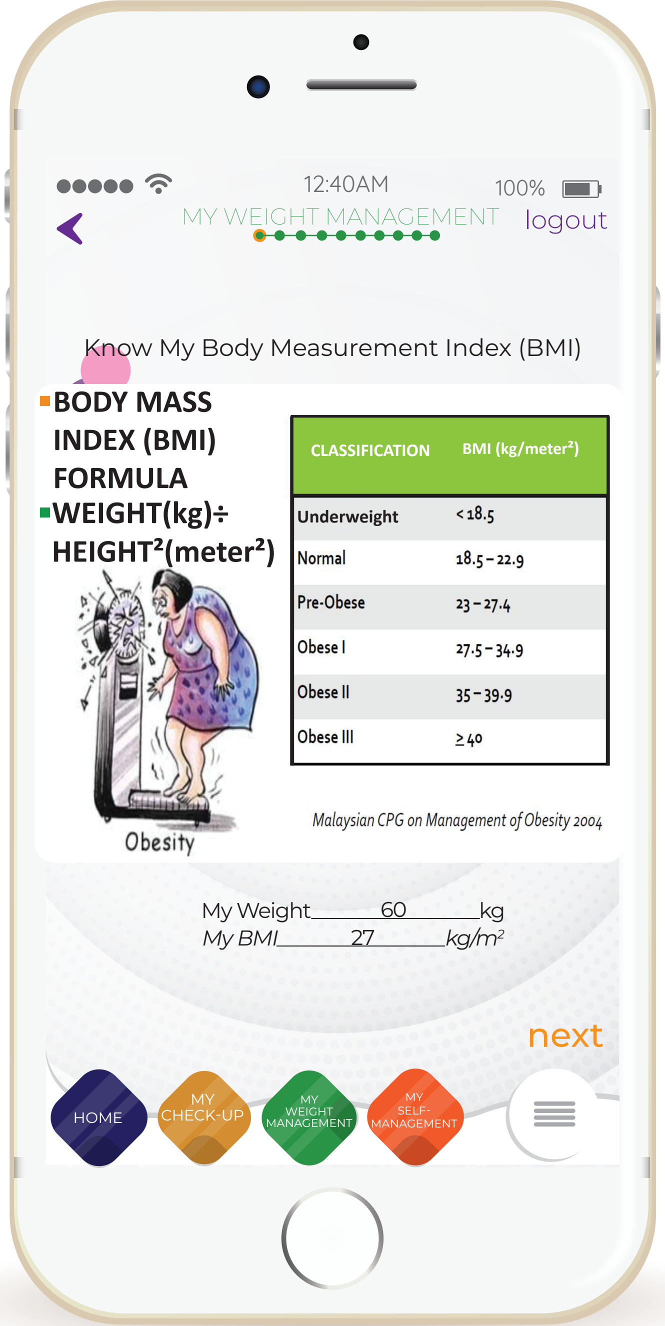

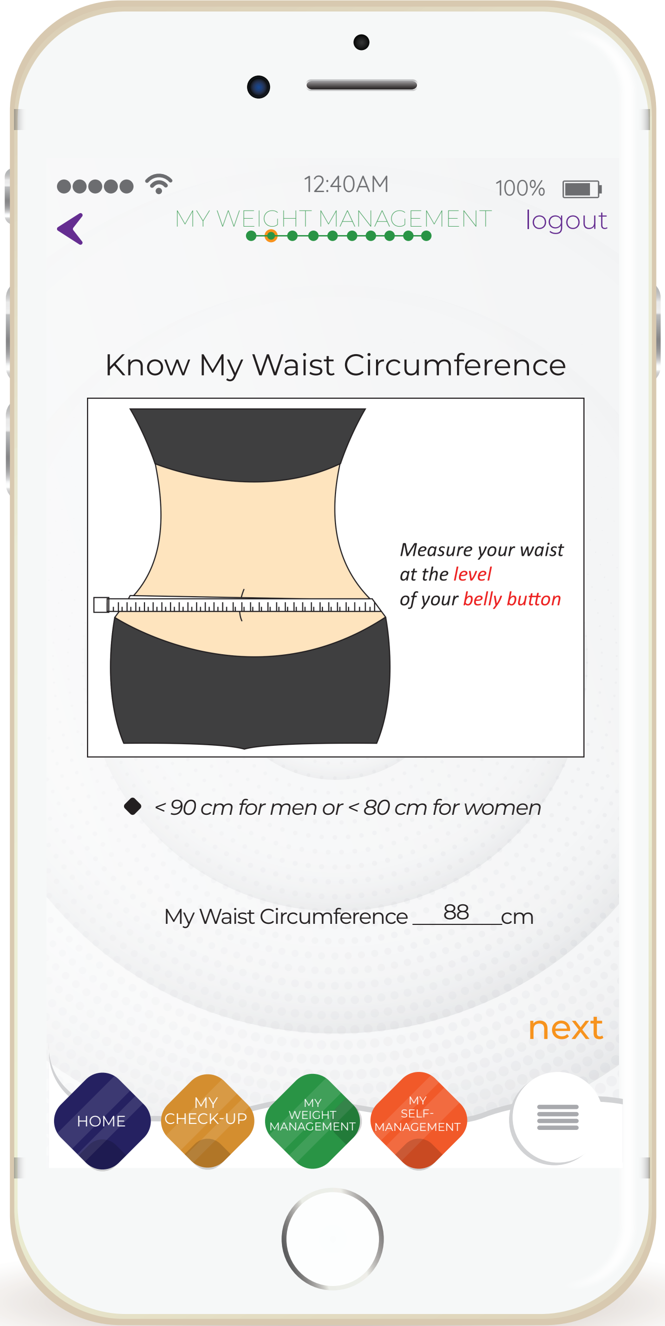

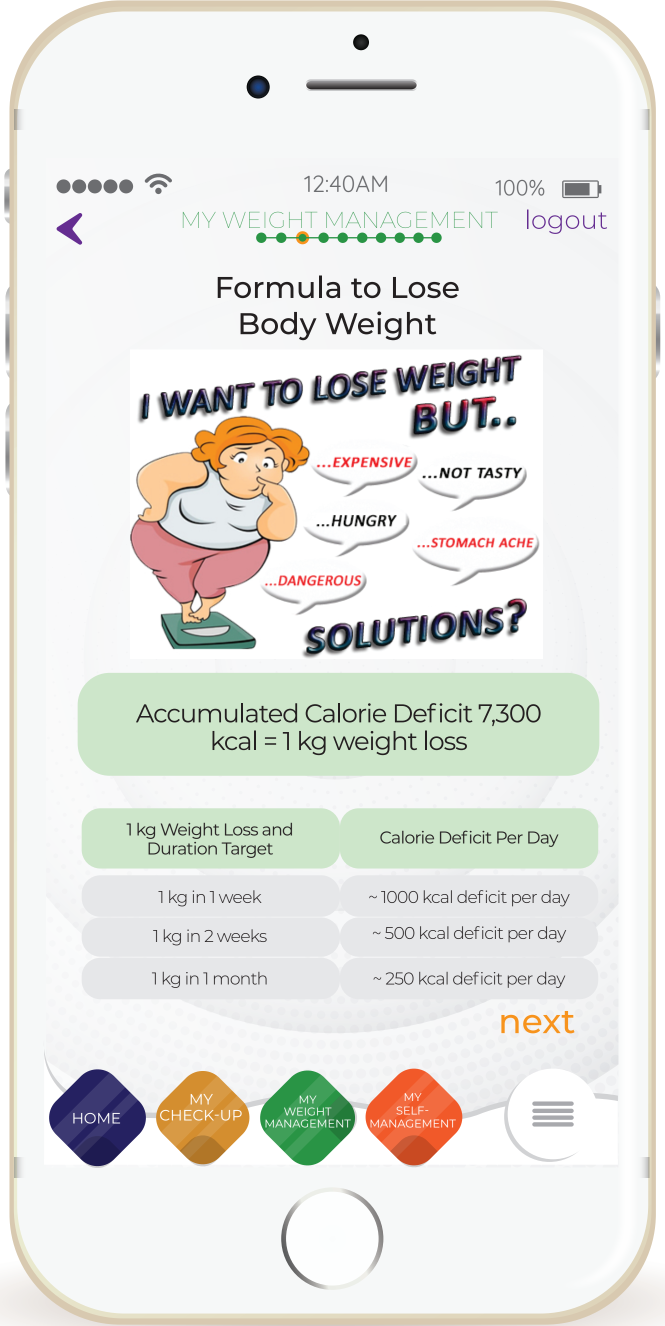
12:40AM
100%
MY WEIGHT MANAGEMENT
logout
Formula to Lose Body Weight
I WANT TO LOSE WEIGHT BUT..
...EXPENSIVE
...NOT TASTY
...HUNGRY
...STOMACH ACHE
...DANGEROUS
SOLUTIONS?
Accumulated Calorie Deficit 7,300 kcal = 1 kg weight loss
1 kg Weight Loss and Duration Target
Calorie Deficit Per Day
1 kg in 1 week
~ 1000 kcal deficit per day
1 kg in 2 weeks
~ 500 kcal deficit per day
1 kg in 1 month
~ 250 kcal deficit per day
next
HOME
MY CHECK-UP
MY WEIGHT MANAGEMENT
MY SELF-MANAGEMENT

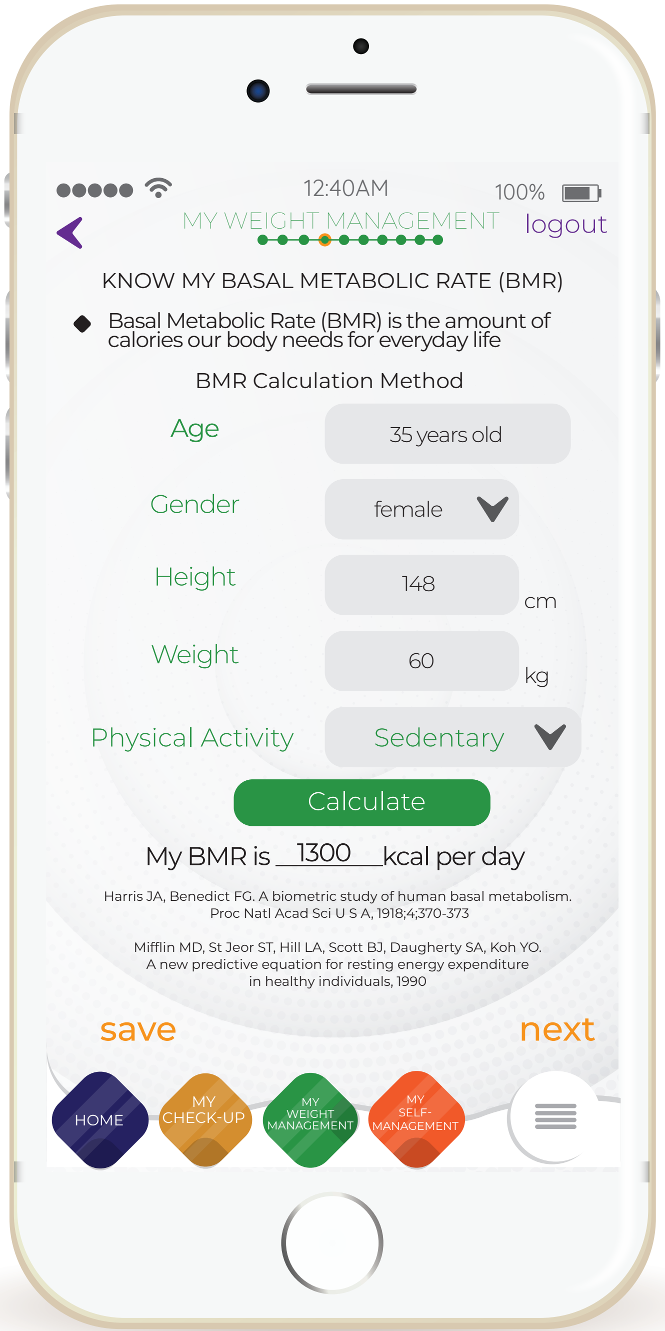
12:40AM
100%
MY WEIGHT MANAGEMENT
logout
KNOW MY BASAL METABOLIC RATE (BMR)
Basal Metabolic Rate (BMR) is the amount of calories our body needs for everyday life
BMR Calculation Method
Age
35 years old
Gender
female
Height
148
cm
Weight
60
kg
Physical Activity
Sedentary
Calculate
My BMR is 1300 kcal per day
Harris JA, Benedict FG. A biometric study of human basal metabolism. Proc Natl Acad Sci U S A, 1918;4;370-373
Mifflin MD, St Jeor ST, Hill LA, Scott BJ, Daugherty SA, Koh YO. A new predictive equation for resting energy expenditure in healthy individuals, 1990
save
next
HOME
MY CHECK-UP
MY WEIGHT MANAGEMENT
MY SELF-MANAGEMENT

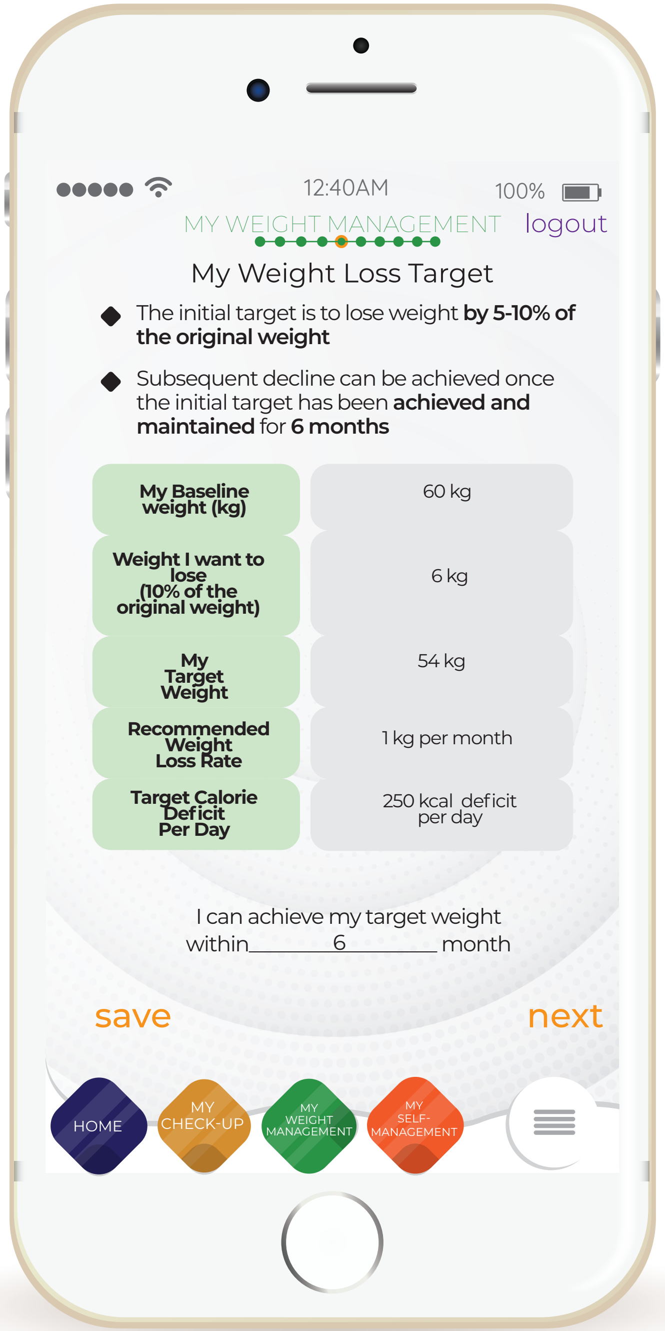
12:40AM
100%
MY WEIGHT MANAGEMENT
logout
My Weight Loss Target
The initial target is to lose weight by 5-10% of the original weight
Subsequent decline can be achieved once the initial target has been achieved and maintained for 6 months
My Baseline weight (kg)
60 kg
Weight I want to lose (10% of the original weight)
6 kg
My Target Weight
54 kg
Recommended Weight Loss Rate
1 kg per month
Target Calorie Deficit Per Day
250 kcal deficit per day
I can achieve my target weight within 6 month
save
next
HOME
MY CHECK-UP
MY WEIGHT MANAGEMENT
MY SELF-MANAGEMENT

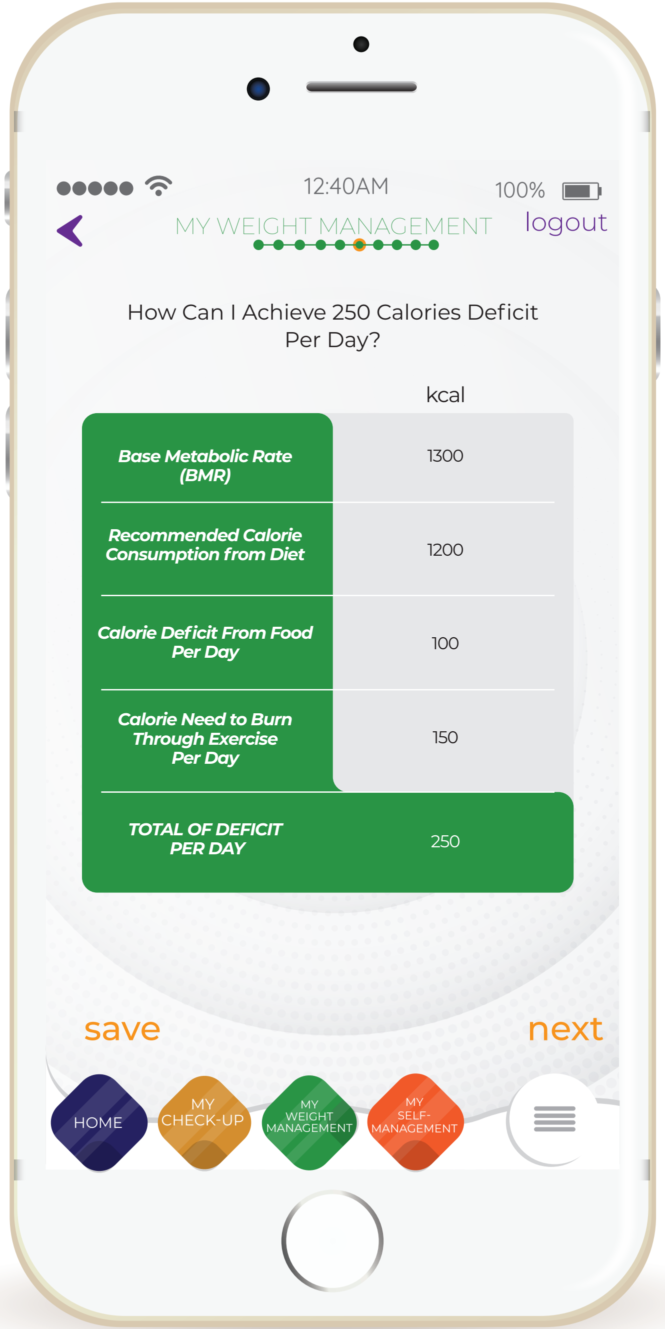
12:40AM
100%
MY WEIGHT MANAGEMENT
logout
How Can I Achieve 250 Calories Deficit Per Day?
kcal
Base Metabolic Rate (BMR)
1300
Recommended Calorie Consumption from Diet
1200
Calorie Deficit From Food Per Day
100
Calorie Need to Burn Through Exercise Per Day
150
TOTAL OF DEFICIT PER DAY
250
save
next
HOME
MY CHECK-UP
MY WEIGHT MANAGEMENT
MY SELF-MANAGEMENT

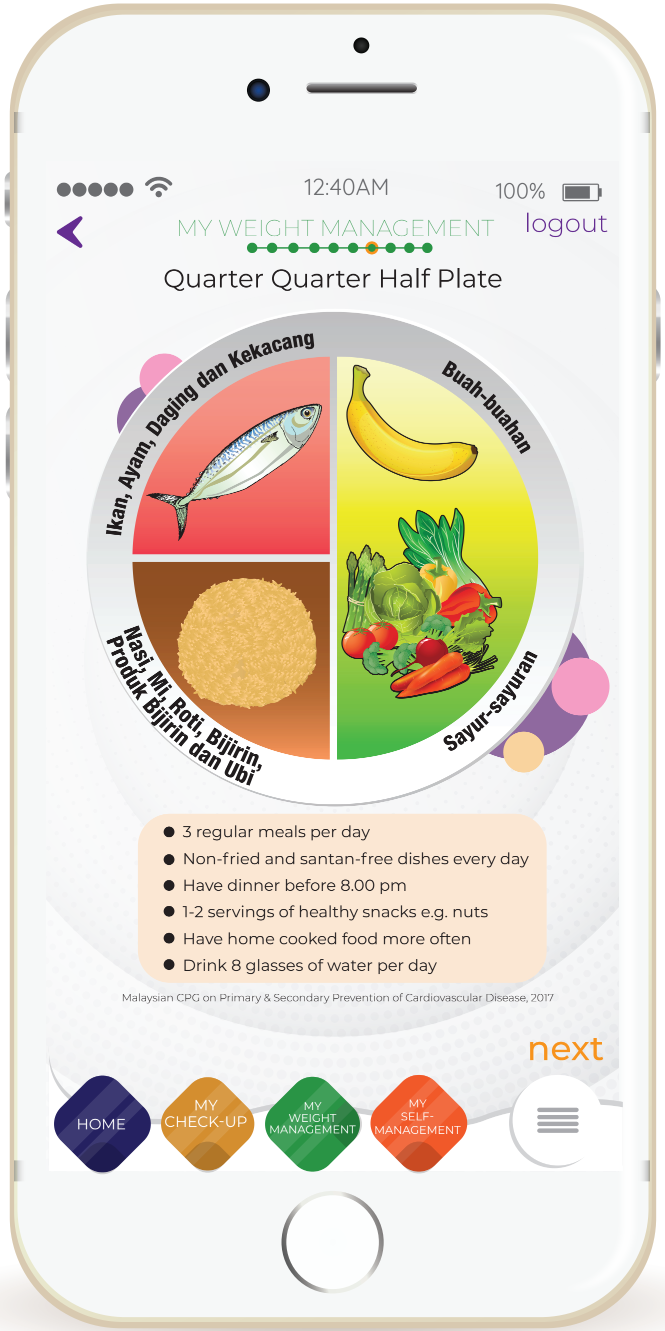
12:40AM
100%
MY WEIGHT MANAGEMENT
logout
Quarter Quarter Half Plate
Ikan, Ayam, Daging dan Kekacang
Buah-buahan
Nasi, Mi, Roti, Bijirin, Produk Bijirin dan Ubi
Sayur-sayuran
3 regular meals per day
Non-fried and santan-free dishes every day
Have dinner before 8.00 pm
1-2 servings of healthy snacks e.g. nuts
Have home cooked food more often
Drink 8 glasses of water per day
Malaysian CPG on Primary & Secondary Prevention of Cardiovascular Disease, 2017
next
HOME
MY CHECK-UP
MY WEIGHT MANAGEMENT
MY SELF-MANAGEMENT

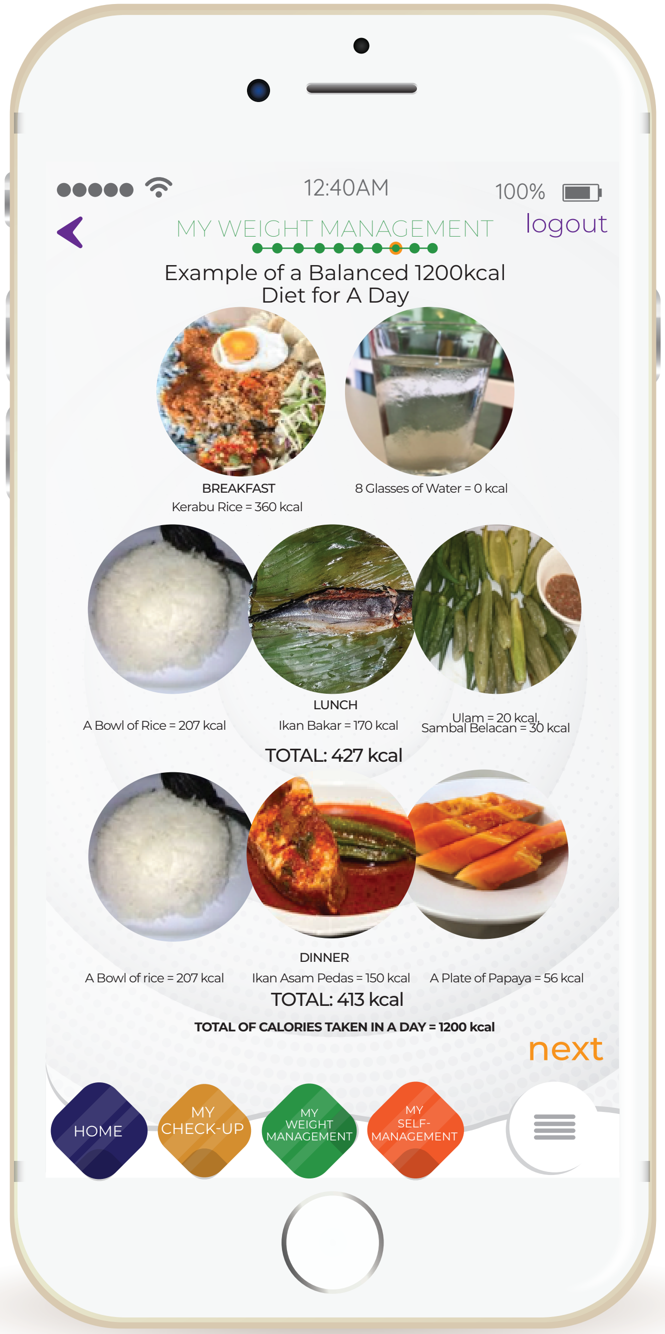
12:40AM
100%
MY WEIGHT MANAGEMENT
logout
Example of a Balanced 1200kcal Diet for A Day
BREAKFAST
Kerabu Rice = 360 kcal
8 Glasses of Water = 0 kcal
LUNCH
A Bowl of Rice = 207 kcal
Ikan Bakar = 170 kcal
Ulam = 20 kcal
Sambal Belacan = 30 kcal
TOTAL: 427 kcal
DINNER
A Bowl of rice = 207 kcal
Ikan Asam Pedas = 150 kcal
A Plate of Papaya = 56 kcal
TOTAL: 413 kcal
TOTAL OF CALORIES TAKEN IN A DAY = 1200 kcal
next
HOME
MY CHECK-UP
MY WEIGHT MANAGEMENT
MY SELF-MANAGEMENT

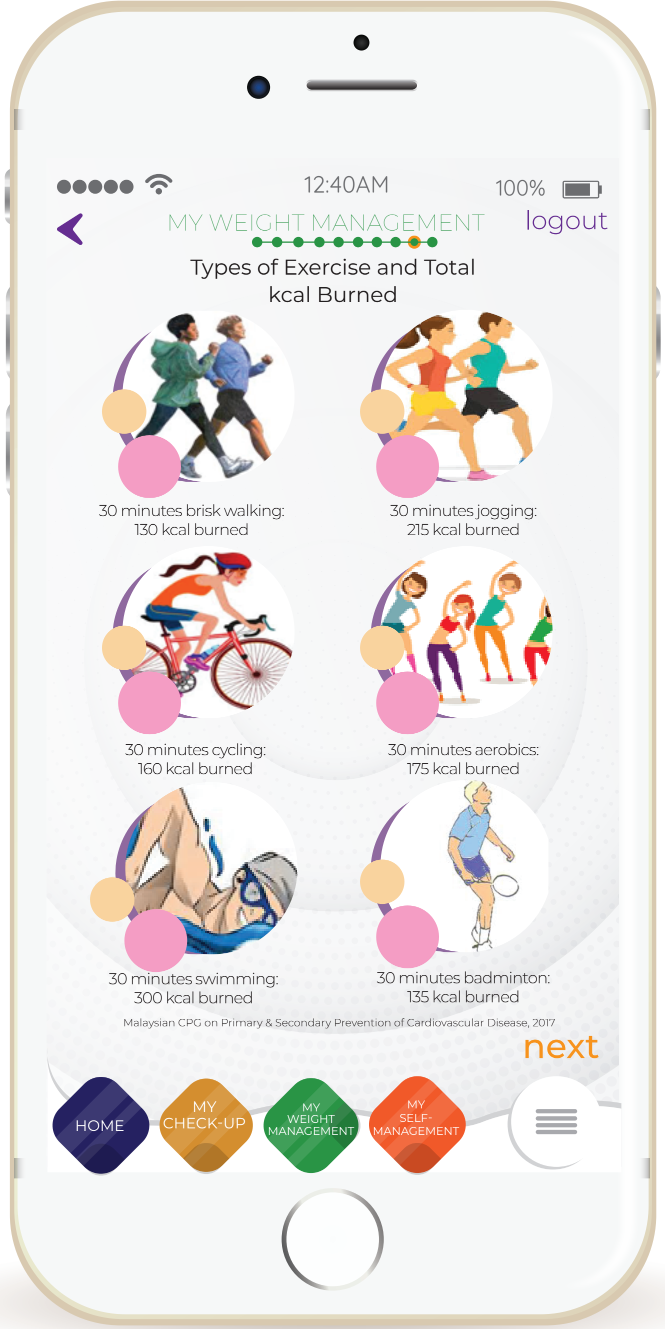
12:40AM
100%
MY WEIGHT MANAGEMENT
logout
Types of Exercise and Total kcal Burned
30 minutes brisk walking: 130 kcal burned
30 minutes jogging: 215 kcal burned
30 minutes cycling: 160 kcal burned
30 minutes aerobics: 175 kcal burned
30 minutes swimming: 300 kcal burned
30 minutes badminton: 135 kcal burned
Malaysian CPG on Primary & Secondary Prevention of Cardiovascular Disease, 2017
next
HOME
MY CHECK-UP
MY WEIGHT MANAGEMENT
MY SELF-MANAGEMENT

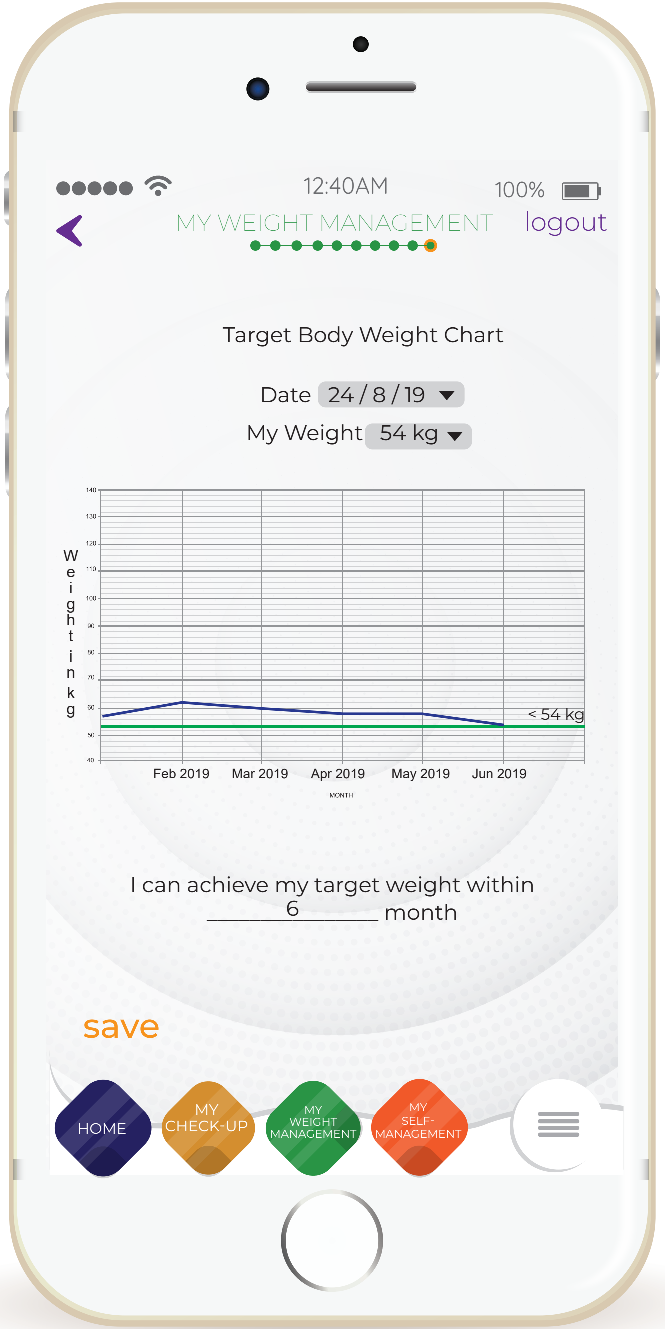
12:40AM
100%
MY WEIGHT MANAGEMENT
logout
Target Body Weight Chart
Date 24 / 8 / 19
My Weight 54 kg
Weight in kg
< 54 kg
Feb 2019
Mar 2019
Apr 2019
May 2019
Jun 2019
MONTH
I can achieve my target weight within 6 month
save
HOME
MY CHECK-UP
MY WEIGHT MANAGEMENT
MY SELF-MANAGEMENT

MY SELF MANAGEMENT

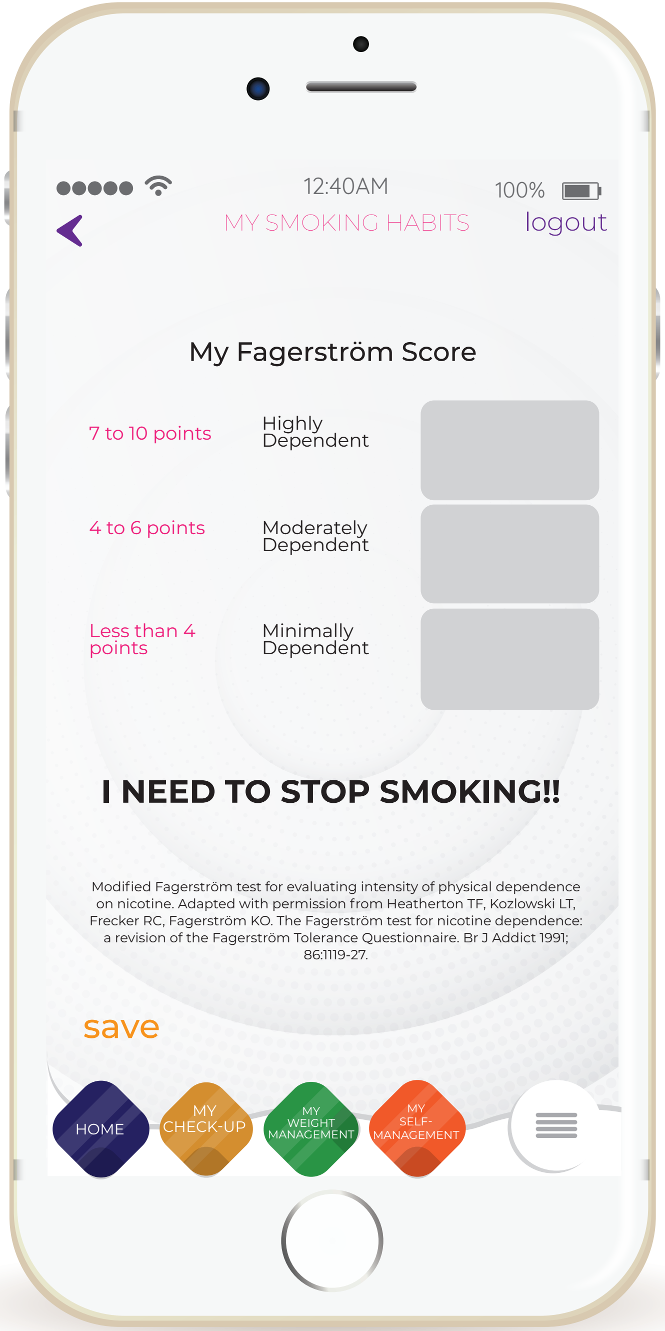

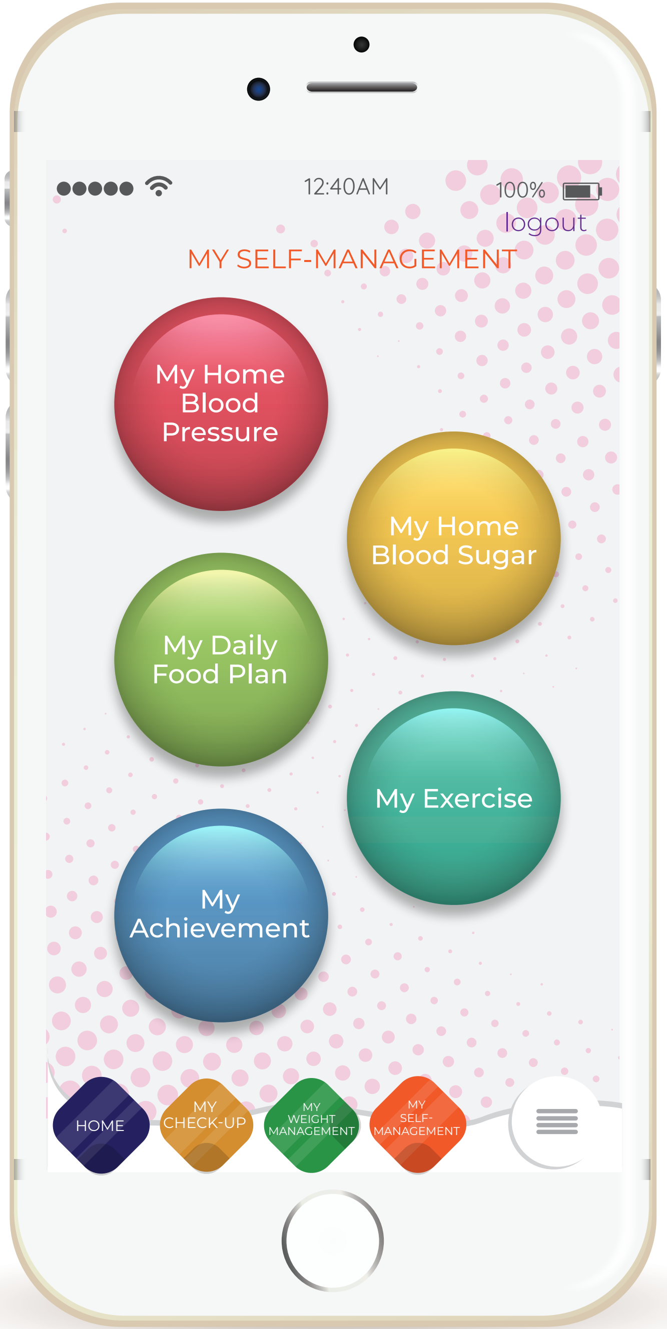

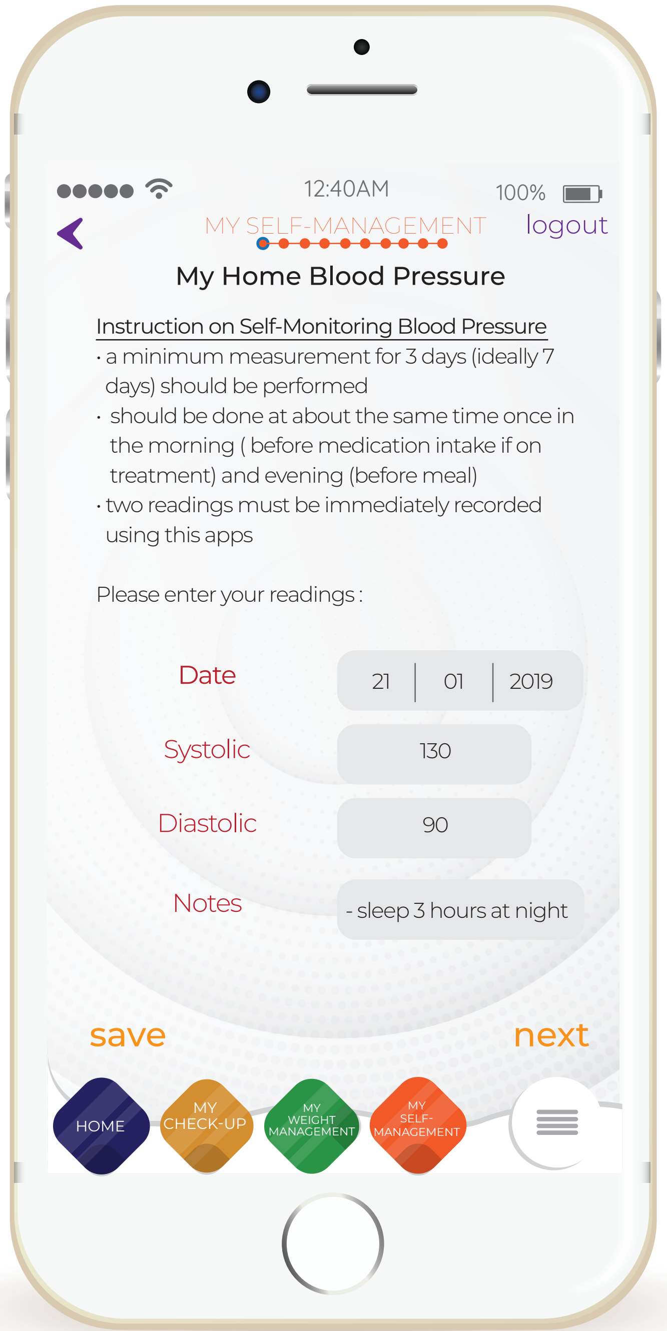

12:40AM
100%
MY SELF-MANAGEMENT
logout
My Home Blood Pressure
Instruction on Self-Monitoring Blood Pressure
· a minimum measurement for 3 days (ideally 7 days) should be performed
· should be done at about the same time once in the morning (before medication intake if on treatment) and evening (before meal)
· two readings must be immediately recorded using this apps
Please enter your readings :
Date
21
01
2019
Systolic
130
Diastolic
90
Notes
- sleep 3 hours at night
save
next
HOME
MY CHECK-UP
MY WEIGHT MANAGEMENT
MY SELF-MANAGEMENT

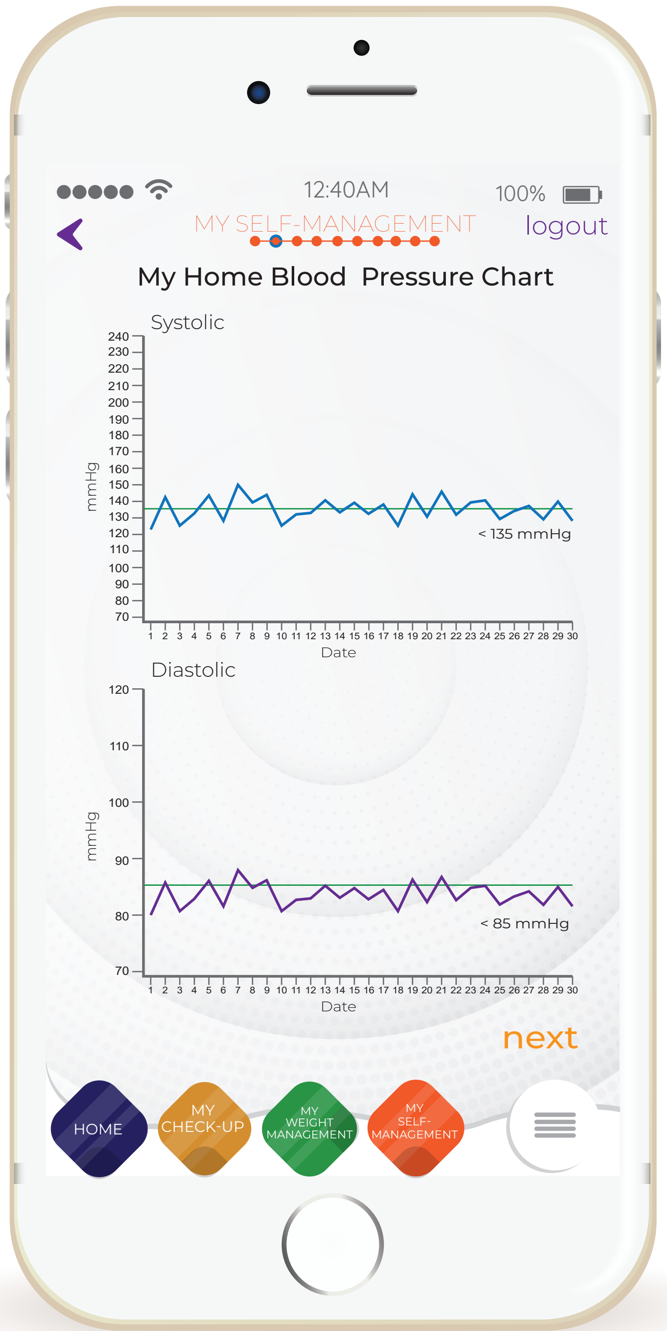

12:40AM
100%
MY SELF-MANAGEMENT
logout
My Home Blood Pressure Chart
Systolic
mmHg
< 135 mmHg
Date
Diastolic
mmHg
< 85 mmHg
Date
next
HOME
MY CHECK-UP
MY WEIGHT MANAGEMENT
MY SELF-MANAGEMENT

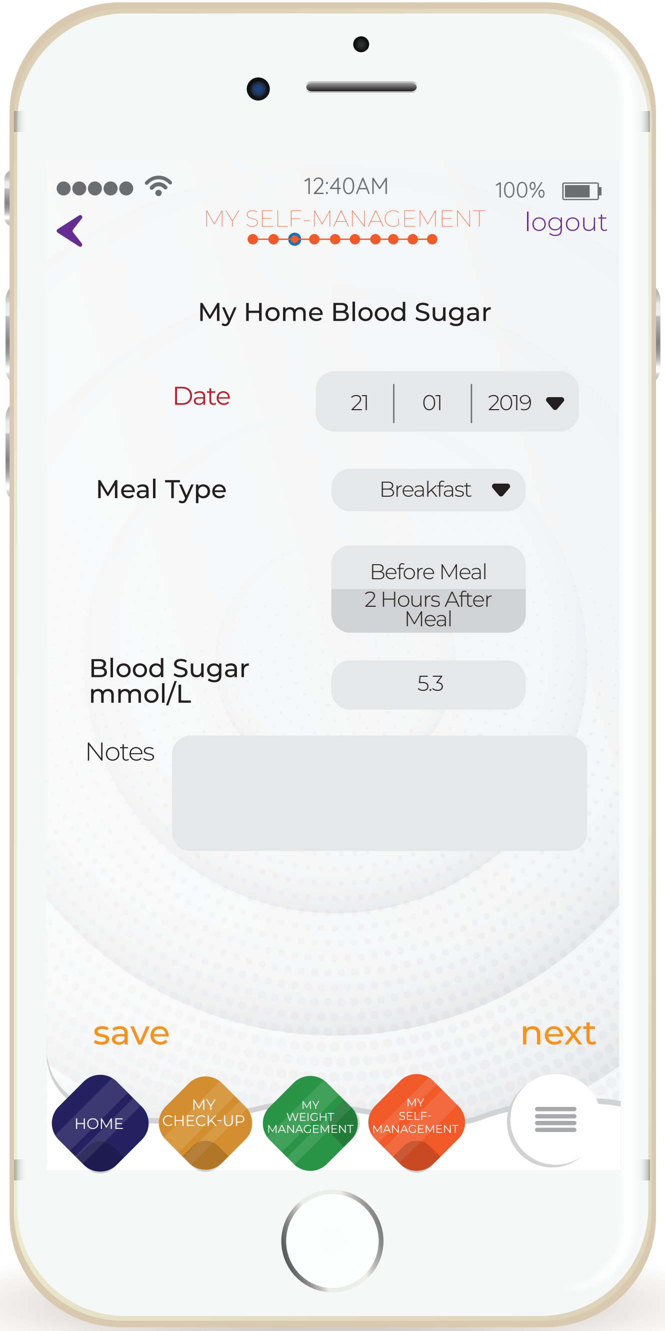
12:40AM
100%
MY SELF-MANAGEMENT
logout
My Home Blood Sugar
Date
21 01 2019
Meal Type
Breakfast
Before Meal
2 Hours After Meal
Blood Sugar mmol/L
5.3
Notes
save
next
HOME
MY CHECK-UP
MY WEIGHT MANAGEMENT
MY SELF-MANAGEMENT

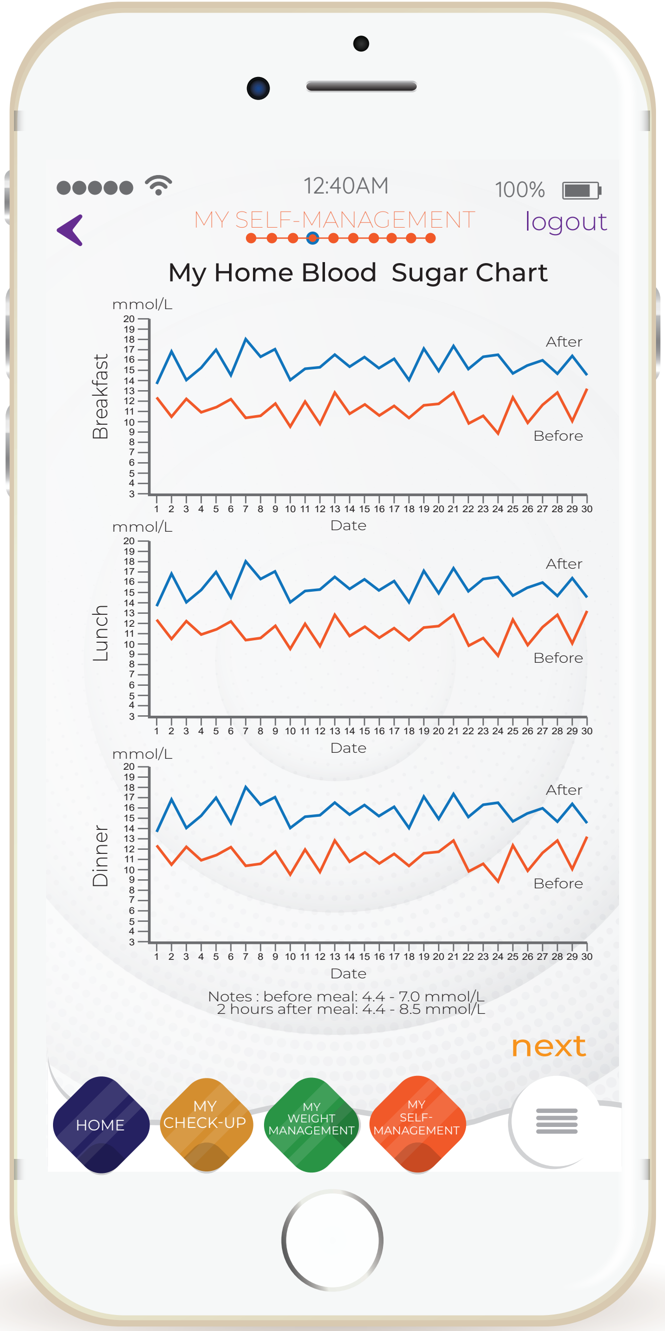
12:40AM
100%
MY SELF-MANAGEMENT
logout
My Home Blood Sugar Chart
mmol/L
Breakfast
After
Before
Date
mmol/L
Lunch
After
Before
Date
mmol/L
Dinner
After
Before
Date
Notes : before meal: 4.4 - 7.0 mmol/L
2 hours after meal: 4.4 - 8.5 mmol/L
next
HOME
MY CHECK-UP
MY WEIGHT MANAGEMENT
MY SELF-MANAGEMENT

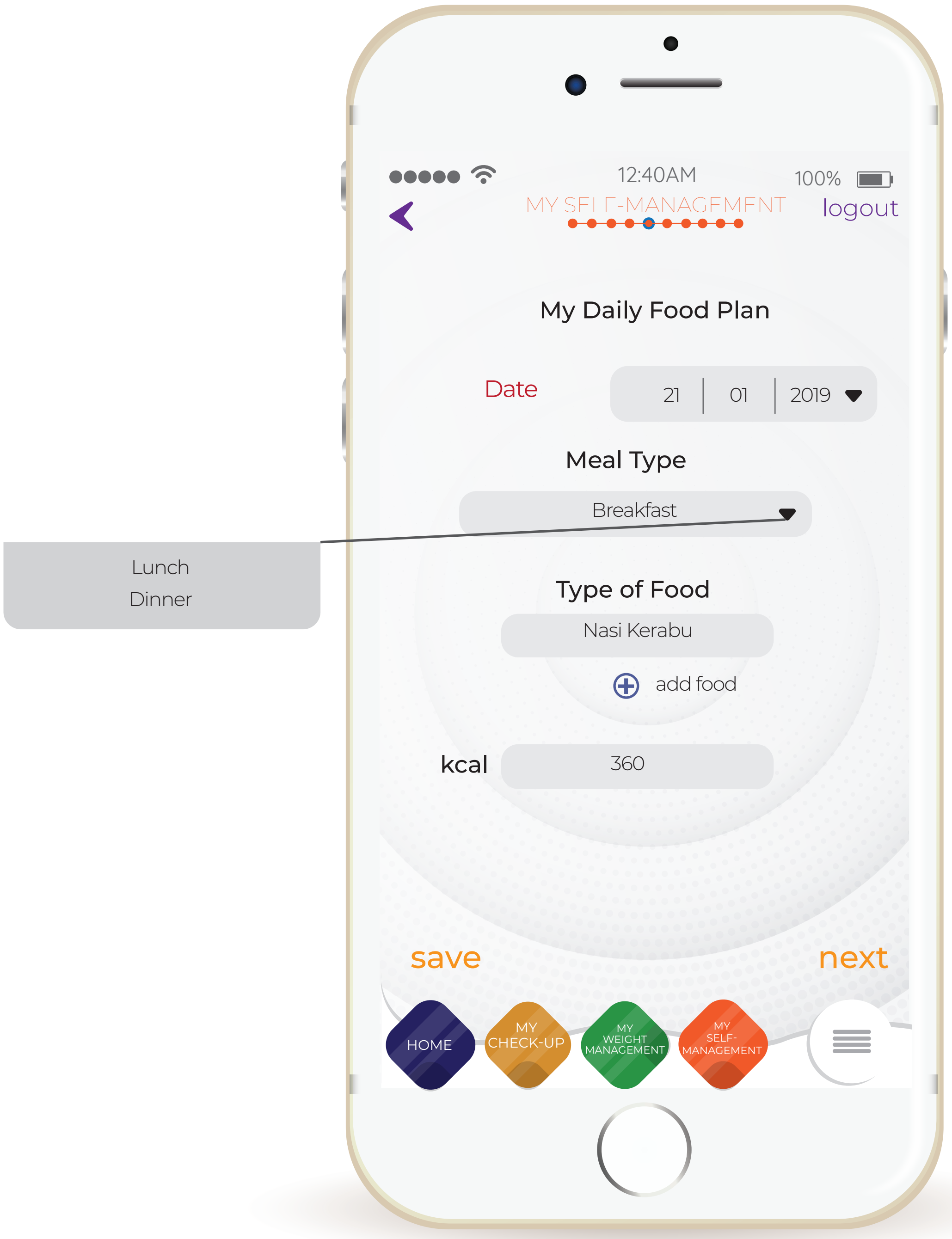
12:40AM
100%
MY SELF-MANAGEMENT
logout
My Daily Food Plan
Date
21 01 2019
Meal Type
Breakfast
Lunch
Dinner
Type of Food
Nasi Kerabu
add food
kcal
360
save
next
HOME
MY CHECK-UP
MY WEIGHT MANAGEMENT
MY SELF-MANAGEMENT

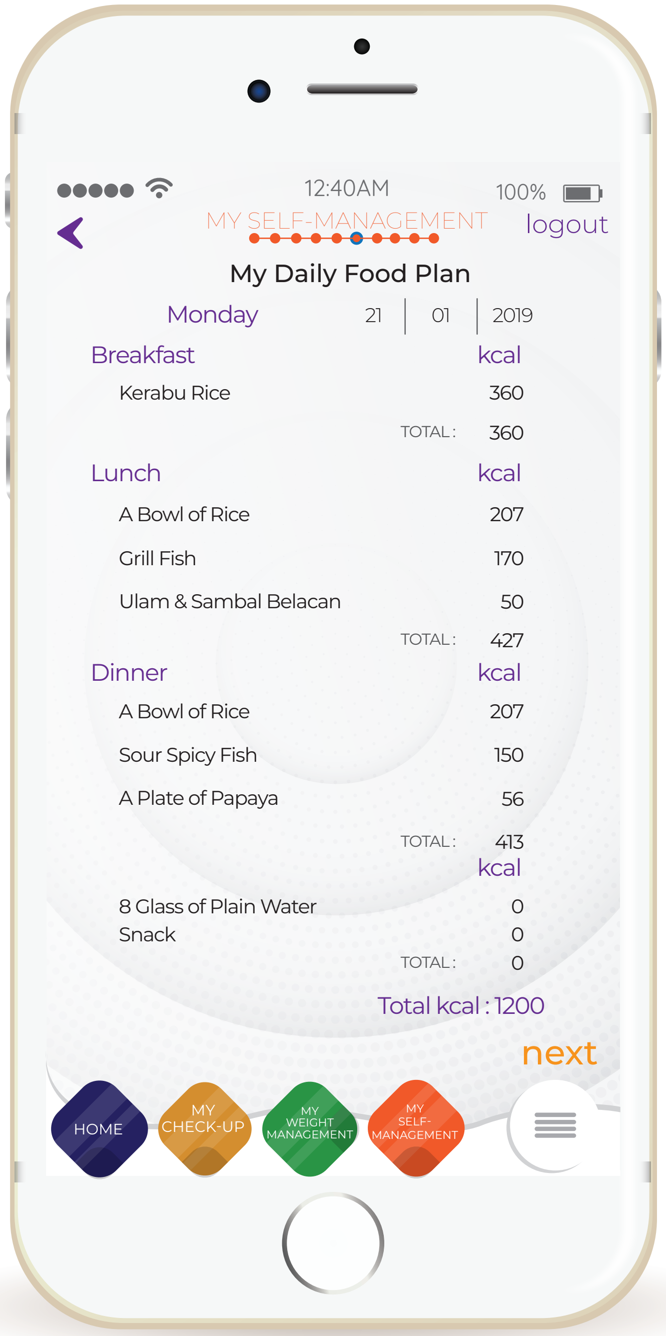
12:40AM
100%
MY SELF-MANAGEMENT
logout
My Daily Food Plan
Monday 21 01 2019
Breakfast kcal
Kerabu Rice 360
TOTAL : 360
Lunch kcal
A Bowl of Rice 207
Grill Fish 170
Ulam & Sambal Belacan 50
TOTAL : 427
Dinner kcal
A Bowl of Rice 207
Sour Spicy Fish 150
A Plate of Papaya 56
TOTAL : 413
kcal
8 Glass of Plain Water 0
Snack 0
TOTAL : 0
Total kcal : 1200
next
HOME
MY CHECK-UP
MY WEIGHT MANAGEMENT
MY SELF-MANAGEMENT

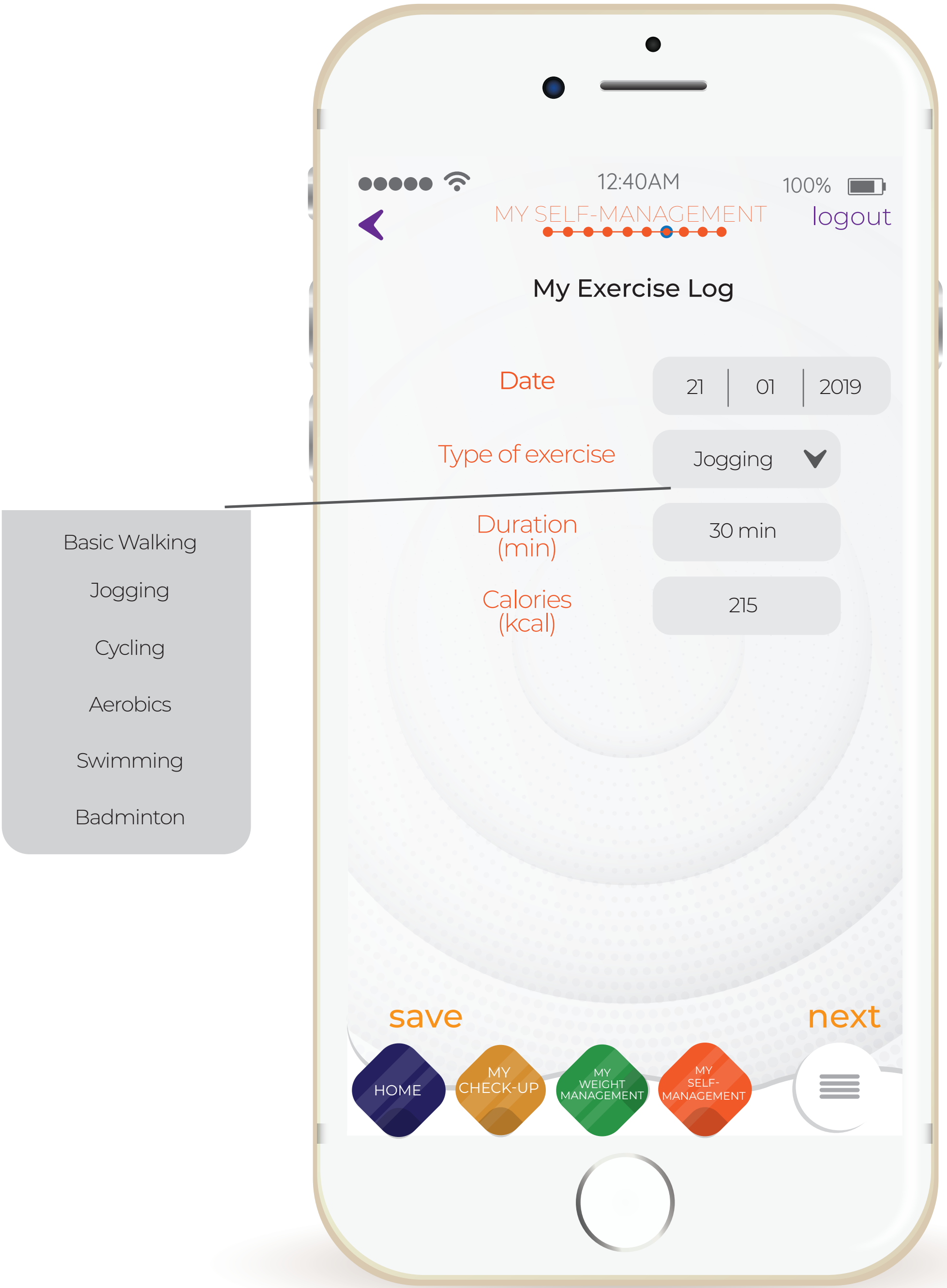
12:40AM
100%
MY SELF-MANAGEMENT
logout
My Exercise Log
Date
21 | 01 | 2019
Type of exercise
Jogging
Duration (min)
30 min
Calories (kcal)
215
Basic Walking
Jogging
Cycling
Aerobics
Swimming
Badminton
save
next
HOME
MY CHECK-UP
MY WEIGHT MANAGEMENT
MY SELF-MANAGEMENT

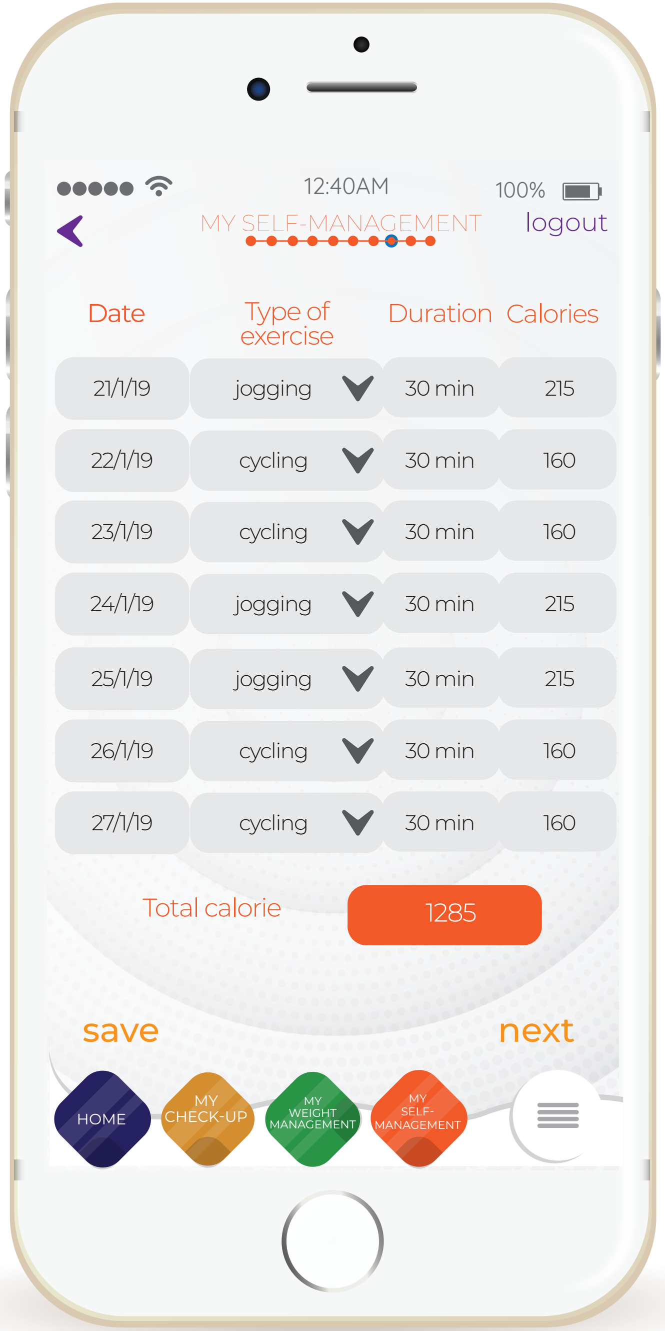
12:40AM
100%
MY SELF-MANAGEMENT
logout
Date
Type of exercise
Duration
Calories
21/1/19 jogging 30 min 215
22/1/19 cycling 30 min 160
23/1/19 cycling 30 min 160
24/1/19 jogging 30 min 215
25/1/19 jogging 30 min 215
26/1/19 cycling 30 min 160
27/1/19 cycling 30 min 160
Total calorie
1285
save
next
HOME
MY CHECK-UP
MY WEIGHT MANAGEMENT
MY SELF-MANAGEMENT

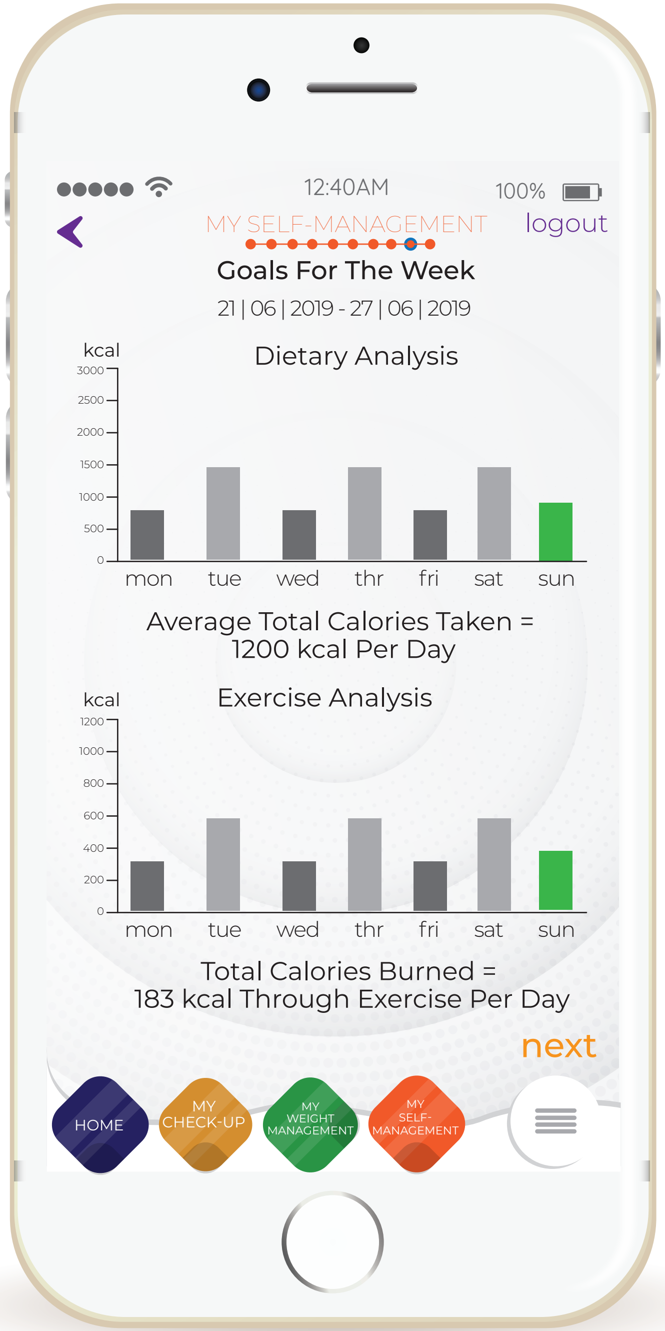
12:40AM
100%
MY SELF-MANAGEMENT
logout
Goals For The Week
21 | 06 | 2019 - 27 | 06 | 2019
kcal
Dietary Analysis
3000
2500
2000
1500
1000
500
0
mon tue wed thr fri sat sun
Average Total Calories Taken =
1200 kcal Per Day
kcal
Exercise Analysis
1200
1000
800
600
400
200
0
mon tue wed thr fri sat sun
Total Calories Burned =
183 kcal Through Exercise Per Day
next
HOME
MY CHECK-UP
MY WEIGHT MANAGEMENT
MY SELF-MANAGEMENT

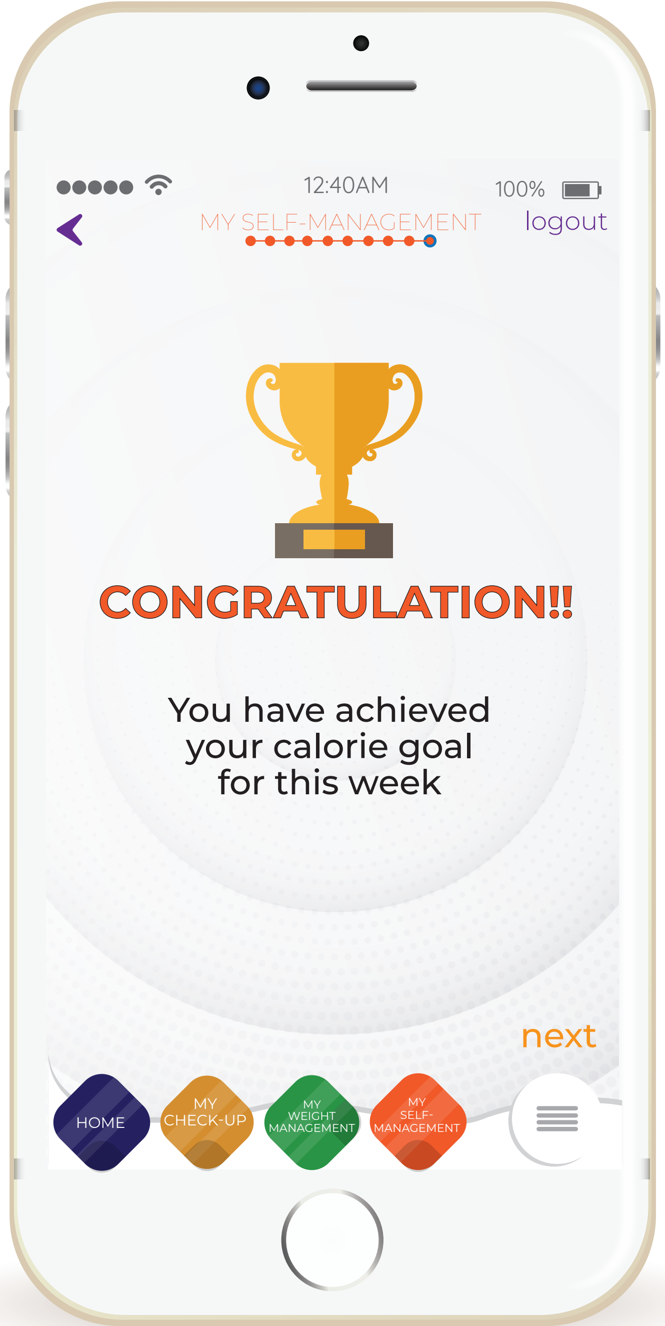
12:40AM
100%
MY SELF-MANAGEMENT
logout
CONGRATULATION!!
You have achieved
your calorie goal
for this week
next
HOME
MY CHECK-UP
MY WEIGHT MANAGEMENT
MY SELF-MANAGEMENT

MY MEDICATION

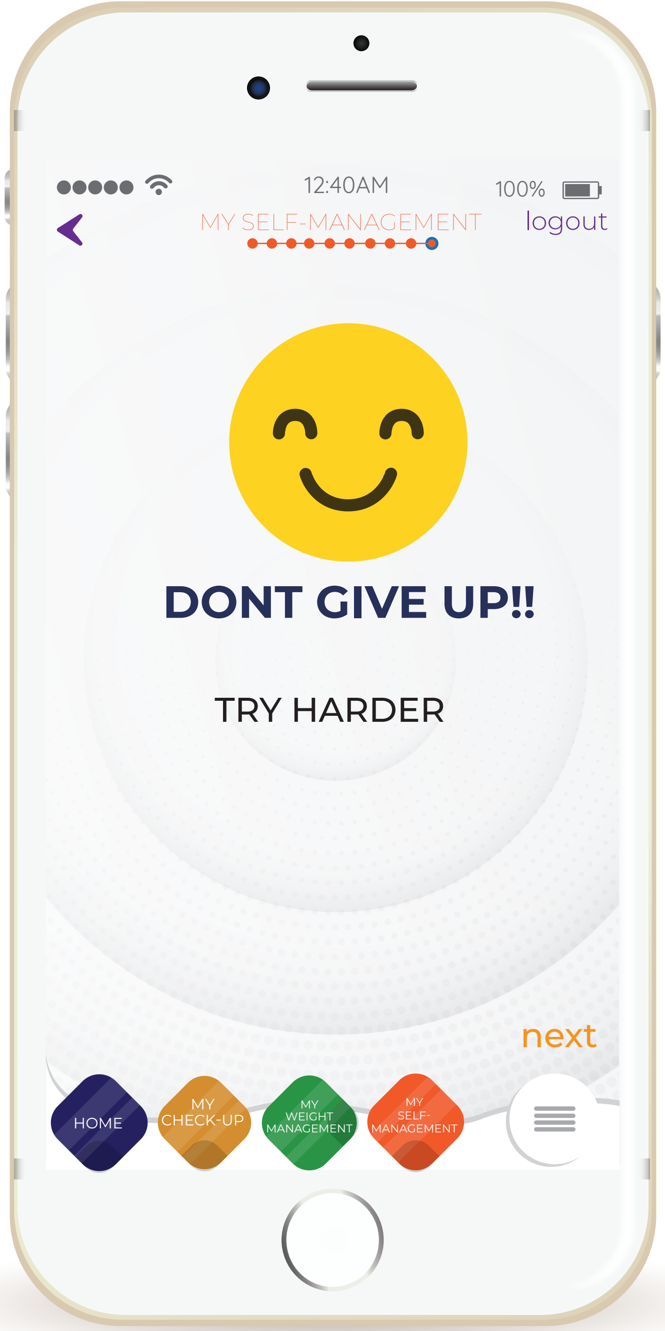

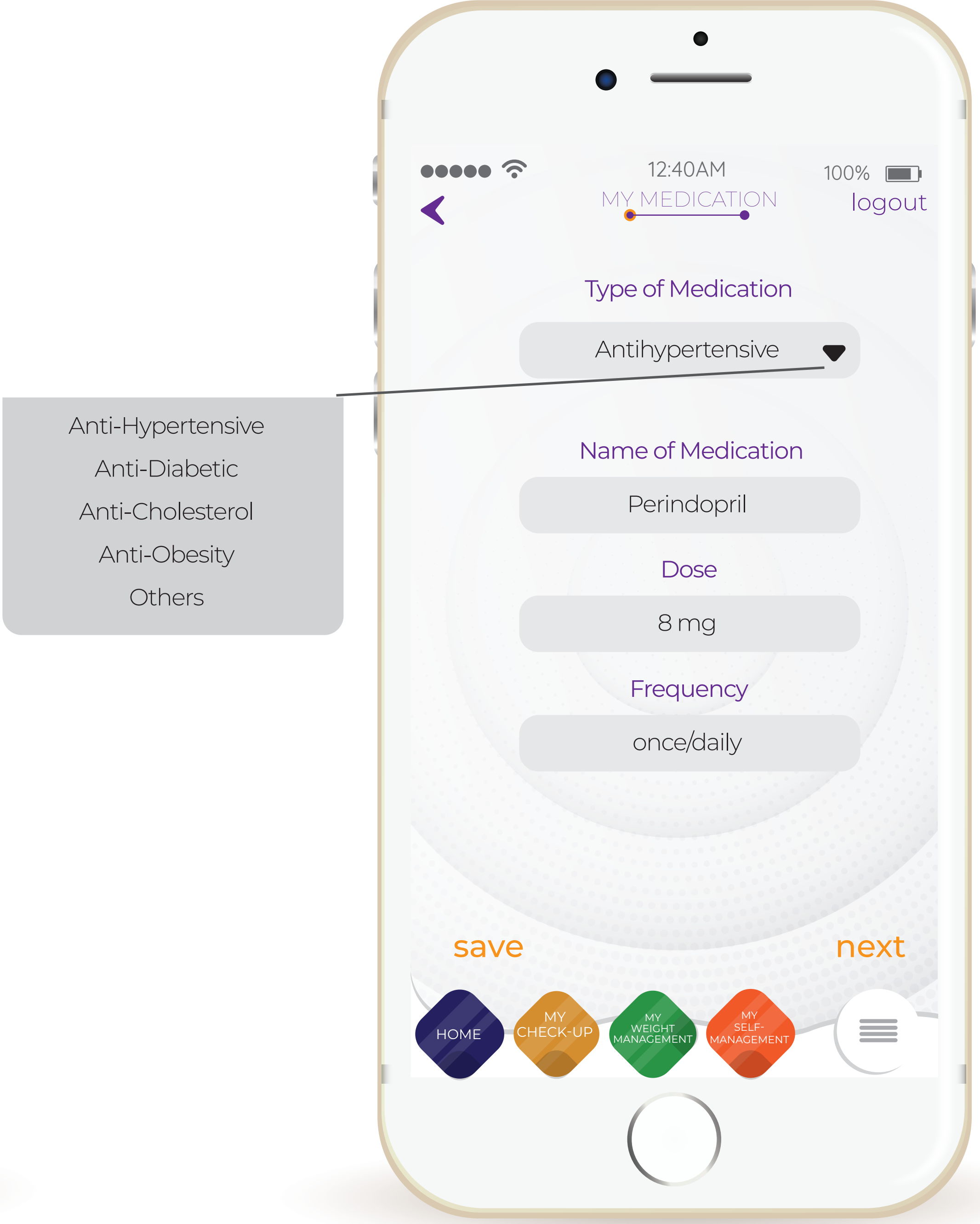

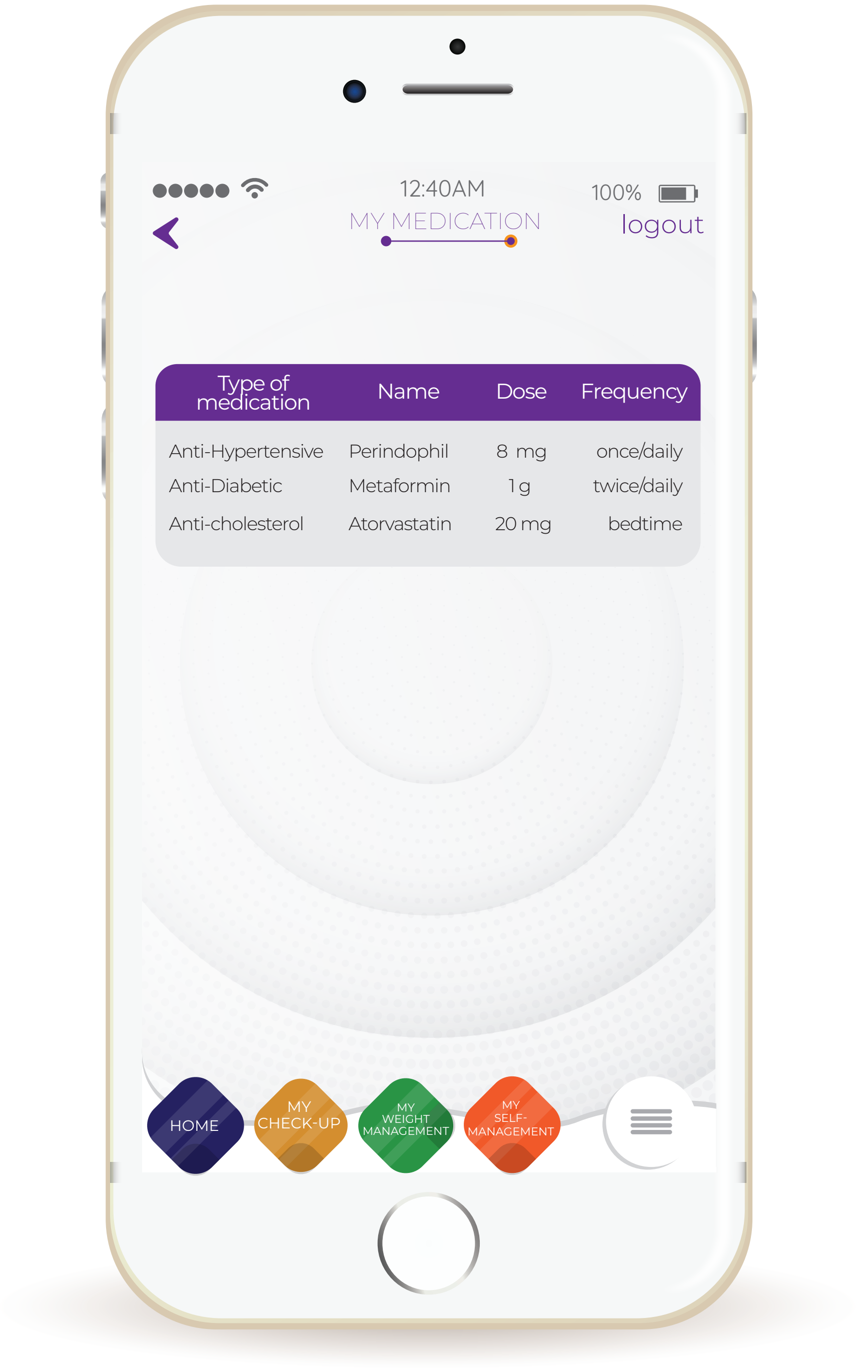
12:40AM
100%
MY MEDICATION
logout
Type of medication
Name
Dose
Frequency
Anti-Hypertensive
Perindophil
8 mg
once/daily
Anti-Diabetic
Metaformin
1 g
twice/daily
Anti-cholesterol
Atorvastatin
20 mg
bedtime
HOME
MY CHECK-UP
MY WEIGHT MANAGEMENT
MY SELF-MANAGEMENT
