# Supplementary material for: The EMPOWER-SUSTAIN e-Health Intervention to improve patient activation and self-management behaviours among individuals with Metabolic Syndrome in primary care: study protocol for a pilot randomised controlled trial
Source: Trials. 2020 Apr 5;21:311. doi: 10.1186/s13063-020-04237-x (PMC7130454; doi:10.1186/s13063-020-04237-x)
Supplement: Supplementary file 3 — Additional file 3. The EMPOWER-SUSTAIN patient consent form. [file 13063_2020_4237_MOESM3_ESM.doc]

**Patient Information Sheet**

**The EMPOWER-SUSTAIN e-Health Intervention to improve patient activation and self-management behaviours among individuals with Metabolic Syndrome in primary care: a pilot randomised controlled trial**

**Purpose of Study**

This study aims to evaluate feasibility and potential effectiveness of the EMPOWER-SUSTAIN mobile application, a self-management tool to improve patient activation and self-management behaviours among individuals with Metabolic Syndrome in the Malaysian primary care setting.

**Study Procedure**

You are invited to participate in this study. If you agree to participate, you will be screened according to several criteria to determine your eligibility. If you are eligible and agree to participate, you will be requested to sign the consent form before participating in the study.

Once you are recruited into the study, you will be interviewed by the researcher on your demographic details and clinical information. We will measure your waist circumference, body mass index and blood pressure. You will then be requested to answer a set of questionnaires which include PAM 13-M, IPAQ-M, DEBQ-M, PACIC-M, SKIP-11 and a visual analogue scale to record the perceived absolute 10-year CVD risk and it should take around 30 minutes to complete.

The researchers will randomly select patients to be in the ‘Intervention’ arm of the study. You will be informed if you are selected to be in the ‘Intervention’ arm.

If you belong to the ‘Intervention’ group,

1. You will be given a username and password to access the EMPOWER-SUSTAIN Self-Management mobile app to use for 6 months and also after the study (if you choose to continue using).
2. You also will be trained on how to use the EMPOWER-SUSTAIN Self-Management mobile app by the doctor. You will be counselled by your doctor on how to change your lifestyle and how to achieve better control of your conditions using the app at home.
3. You will be requested to attend follow-up visits at 3-month and 6-month so that the doctor can monitor your conditions.

If you belong to the control group, you will be followed up by the doctor with usual care as given to all patients. You will be asked to return to the clinic in 6 month time to answer the questionnaires again.

Verbal information about the study will be given by the research assistant if you need further information.

**Participation in Study**

Your participation in this study is entirely voluntary. You may refuse to take part in the study or you may withdraw yourself from participation in the study at any time without penalty.

**Benefit of Study**

The expected benefit of the study is to produce research evidence on potential effectiveness of the EMPOWER-SUSTAIN Self-Management mobile app. Once this study is completed, this app will be made available to all patients attending the UiTM Primary Care Clinic.

If you have any enquiries on the study or your rights, please contact the Principal Investigator, Professor Dr Anis Safura Ramli at 019-384 4503 or e-mail: rossanis_yuzadi@[yahoo.co.uk](mailto:demure519@yahoo.com)

**Confidentiality**

Your medical information will be kept confidential by the investigators at all time and will not be made public unless disclosure is required by law.

By signing this consent form, you will authorize the review of your medical records, analysis and use of the anonymized data for reports and publications arising from this study.

____**___________________________________________________________________** Consent Form

If you agree and are eligible to become a participant in this study, you are requested to sign this Consent Form.

I herewith confirm that I have met the requirement of age and am capable of acting on behalf of myself as follows:

1. I understand the nature and scope of the research being undertaken.

2. I have read and understood all the terms and conditions of my participation in the research.

3. All my questions relating to this research and my participation therein have been answered to my satisfaction.

4. I voluntarily agree to take part in this research, to follow the study procedures and to provide all necessary information to the investigators as requested.

5. I may at any time choose to withdraw from this research without giving reasons.

6. I have received a copy of the Patient Information Sheet and Consent Form.

7. Except for damages resulting directly from negligent or malicious conduct of the researcher(s), I hereby release and discharge UiTM and all participating researchers from all unintended liability which may or may not be associated with or related to my participation and agree to hold them harmless from any harm or loss that may be incurred by me due to my participation in the research.

________________________________________________________________________Name of Participant Signature

________________________________________________________________________I.C No Date

________________________________________________________________________Name of Witness Signature

________________________________________________________________________I.C No Date

________________________________________________________________________Name of Consent Taker Signature

________________________________________________________________________I.C No Date
