# Supplementary material for: The EMPOWER-SUSTAIN e-Health Intervention to improve patient activation and self-management behaviours among individuals with Metabolic Syndrome in primary care: study protocol for a pilot randomised controlled trial
Source: Trials. 2020 Apr 5;21:311. doi: 10.1186/s13063-020-04237-x (PMC7130454; doi:10.1186/s13063-020-04237-x)
Supplement: Supplementary file 4 — Additional file 4. The EMPOWER-SUSTAIN physician consent form. [file 13063_2020_4237_MOESM4_ESM.doc]

**Physician/Nurse Information Sheet**

**The EMPOWER-SUSTAIN e-Health Intervention to improve patient activation and self-management behaviours among individuals with Metabolic Syndrome in primary care: a pilot randomised controlled trial**

**Purpose of Study**

This study aims to evaluate feasibility and potential effectiveness of the EMPOWER-SUSTAIN mobile application, a self-management tool to improve patient activation and self-management behaviours among individuals with Metabolic Syndrome in the Malaysian primary care setting.

**Study Procedure**

You are invited to participate in this study. If you agree to participate, you will be screened according to several criteria to determine your eligibility. If you are eligible and agree to participate, you will be requested to sign the consent form before participating in the study.

You will be informed if you are selected to be in the ‘Intervention’ arm of the study.

As the Primary Care Physician (PCP) or nurse participating in the ‘Intervention’ arm of this study:

1. You will be trained in the EMPOWER-SUSTAIN training workshop on how to utilise the EMPOWER-SUSTAIN Self-Management web-based desktop and mobile applications.
2. You will be involved in delivering the EMPOWER-SUSTAIN eHealth Intervention to patients in the intervention arm for 6 months.
3. You will arrange follow-up in the EMPOWER-SUSTAIN Clinic at baseline, 3-month and 6-month for patients with MetS in the intervention group.

If you are in the ‘Control’ arm, you will continue with usual care in managing your patients.

**Participation in Study**

Your participation in this study is entirely voluntary. You may refuse to take part in the study or you may withdraw yourself from participation in the study at any time without penalty.

**Benefit of Study**

The expected benefit of the study is to produce research evidence on potential effectiveness of the EMPOWER-SUSTAIN Self-Management mobile app. Once this study is completed, this app will be made available to all patients attending the UiTM Primary Care Clinic.

If you have any enquiries on the study or your rights, please contact the primary investigator, Professor Dr Anis Safura Ramli at 019-384 4503 or e-mail: rossanis_yuzadi@[yahoo.co.uk](mailto:demure519@yahoo.com)

**Confidentiality**

Your patients’ medical information will be kept confidential by the investigators at all time and will not be made public unless disclosure is required by law.

By signing this consent form, you will authorize the review of your patients’ medical records, analysis and use of the anonymized data for reports and publications arising from this study.

____**___________________________________________________________________** Consent Form

If you agree to become a participating PCP or nurse in this study, you are required to sign this Consent Form.

I herewith confirm that I have met the requirement of age and am capable of acting on behalf of myself as follows:

1. I understand the nature and scope of the research being undertaken.

2. I have read and understood all the terms and conditions of my participation in the research.

3. All my questions relating to this research and my participation therein have been answered to my satisfaction.

4. I voluntarily agree to take part in this research, to follow the study procedures and to provide all necessary information to the investigators as requested.

5. I may at any time choose to withdraw from this research without giving reasons.

6. I have received a copy of the Physician/Nurse Information Sheet and Consent Form.

7. Except for damages resulting directly from negligent or malicious conduct of the researcher(s), I hereby release and discharge UiTM and all participating researchers from all unintended liability which may or may not be associated with or related to my participation and agree to hold them harmless from any harm or loss that may be incurred by me due to my participation in the research.

________________________________________________________________________Name of Physician/Nurse Signature

________________________________________________________________________I.C No Date

________________________________________________________________________Name of Witness Signature

________________________________________________________________________I.C No Date

________________________________________________________________________Name of Consent Taker Signature

________________________________________________________________________I.C No Date
